# Administration of macrolide antibiotics increases cardiovascular risk

**DOI:** 10.3389/fcvm.2023.1117254

**Published:** 2023-02-23

**Authors:** Yang Wu, Wen-Tao Bi, Li-Ping Qu, Jun Fan, Xiang-Jun Kong, Cheng-Cheng Ji, Xu-Miao Chen, Feng-Juan Yao, Li-Juan Liu, Yun-Jiu Cheng, Su-Hua Wu

**Affiliations:** ^1^Department of Cardiology, The First Affiliated Hospital, Sun Yat-sen University, Guangzhou, China; ^2^NHC Key Laboratory of Assisted Circulation, Sun Yat-sen University, Guangzhou, China; ^3^Department of Cardiology, Guangzhou First People’s Hospital, School of Medicine, South China University of Technology, Guangzhou, China; ^4^Department of Medical Ultrasonics, The First Affiliated Hospital, Sun Yat-sen University, Guangzhou, China; ^5^Department of Cardiology, Guangdong Cardiovascular Institute, Guangdong Provincial People’s Hospital, Guangdong Academy of Medical Sciences, Guangzhou, China

**Keywords:** sudden cardiac death, macrolide antibiotics, cardiovascular risk, meta-analysis, ventricular arrhythmia

## Abstract

**Background:**

The increased risk of cardiovascular events in patients prescribed macrolides has been subject to debate for decades.

**Methods:**

Medline, EMBASE databases and ClinicalTrials.gov were searched from inception until August 31, 2022 for studies investigating the link between macrolides and cardiovascular risk. A meta-analysis was performed using a random-effects model.

**Results:**

A total of 80 studies involving 39,374,874 patients were included. No association was found between macrolides and all-cause death. However, compared with the non-macrolide group, macrolides were associated with a significantly increased risk of ventricular arrhythmia or sudden cardiac death (VA or SCD) (azithromycin, relative ratio [RR]: 1.53; 95% confidence interval [CI]: 1.19 to 1.97; clarithromycin, RR: 1.52; 95% CI: 1.07 to 2.16). Besides, administration of macrolides was associated with a higher risk of cardiovascular disease (CVD) death (azithromycin, RR: 1.63; 95% CI: 1.17 to 2.27) and a slightly increased risk of myocardial infarction (MI) (azithromycin, RR: 1.08; 95% CI: 1.02 to 1.15). Interestingly, no association was observed between roxithromycin and adverse cardiac outcomes. Increased risk of VA or SCD was observed for recent or current use of macrolides, MI for former use, and CVD death for current use.

**Conclusion:**

Administration of macrolide antibiotics and timing of macrolide use are associated with increased risk for SCD or VTA and cardiovascular death, but not all-cause death.

## Introduction

Macrolide antibiotics represent an important class of orally active antibiotics that act as bacteriostatic agents, including azithromycin, erythromycin and clarithromycin. Among these, azithromycin is the most widely prescribed, with 51.5 million prescriptions in the United States in 2013 (1). It has long been thought that macrolide antibiotics are associated with cardiovascular events including myocardial infarction (MI), ventricular tachyarrhythmias, and sudden cardiac death (SCD) (2, 3).

Our previous study substantiated that macrolide antibiotics increased the risk of SCD/ventricular tachyarrhythmia and cardiovascular death, but not all-cause mortality (3). However, one recent study showed that macrolide antibiotics are associated with a significant risk for MI but not arrhythmia and cardiovascular mortality (4). Another study indicated that macrolide use did not increase the risk of ventricular arrhythmias in older patients. Interestingly, macrolide antibiotics were associated with a lower risk of all-cause mortality (5). These findings highlight the controversy surrounding warnings issued by the US Food and Drug Administration (6). Current evidence suggests Coronavirus Disease 2019 (COVID-19) is closely related to cardiovascular disease (CVD). Accordingly, it is of great significance to explore whether macrolides lead to cardiovascular complications, especially during the treatment of COVID-19 patients (7).

In recent years, much emphasis has been placed on the safety of macrolides in patients with CVD. Indeed, patient safety and prevention of cardiovascular events should be highlighted when macrolides are administered to patients with COVID-19.

Consequently, it is essential to re-evaluate the association between macrolides and CVD. This meta-analysis brings together the latest evidence, providing a comprehensive update of the relationship between macrolides and CVD risk.

## Materials and methods

### Search strategy

This meta-analysis was registered in PROSPERO (CRD42021260724). We followed the Preferred Reporting Items for Systematic Review and Meta-Analyses (PRISMA) guidelines for this meta-analysis. Two independent investigators (Yang Wu and Wen-Tao Bi) performed the literature search using Medline, EMBASE databases and ClinicalTrials.gov. The computer-based searches included the keywords “macrolides,” “azithromycin,” “erythromycin,” “clarithromycin,” “roxithromycin,” “cardiac,” “heart,” “cardiovascular,” “death,” “stroke,” “myocardial infarction,” “acute coronary syndrome,” “ventricular tachycardia,” “arrhythmia,” “sudden cardiac death,” “cardiac arrest,” “torsades de pointes,” “sudden death,” and “mortality.”

### Study selection

Study selection, data extraction, and data analysis were performed in accordance with the Cochrane Collaboration and the PRISMA guidelines (8). Studies like cohort studies, case-control studies, or randomized controlled trials that reported the association between macrolides and the risk of cardiovascular events were included. For multiple reports of the same study, only the most comprehensive and recent study was included, and the remaining were excluded.

### Data extraction

Two trained reviewers (Yang Wu and Wen-Tao Bi) independently extracted and collected the data from the included studies and assessed the risk of bias. The following data were extracted: source of participants, country, type of study, baseline diseases, type of antibiotics, doses of antibiotics, duration of antibiotics and outcomes. The information recorded for each study is detailed in the supplement. Disagreements between the 2 reviewers regarding extracted data were resolved by consensus. For cohort and case-control studies, the Newcastle-Ottawa scale was used to assess the risk of bias to make the process clearer and more accurate. The modified Jadad score was used to evaluate the quality of RCTs.

### Outcomes

The outcomes were ventricular arrhythmia or sudden cardiac death (VA or SCD), MI, CVD death and all-cause death. The VA or SCD was defined according to the 10th revision of the International Classification of Diseases as ventricular tachycardia, torsades de pointes, ventricular fibrillation, ventricular flutter, sudden cardiac arrest, and SCD.

### Statistical analysis

Our meta-analysis was performed using STATA (version 15.0, Stata Corp., College Station, TX) software package. The random-effects model (Mantel-Haenszel method) was used for analysis. The RR with 95% CI was determined for dichotomous variables. Heterogeneity was assessed using the inconsistency index (I2-statistic) and divided into low (≤ 25%), moderate (> 25% – 50%), and high (> 50%) (9). We also performed a sensitivity analysis setting the endpoint to SCD. Subgroup analyses were performed by stratifying according to drug subtype and timing. The definition of “time of exposure to macrolides” was as follows: current referred to the patient’s current use of macrolides, recent was defined as use of macrolides within one month, and former involved the use of macrolides within one year. Publication bias was assessed with funnel plots using Begg’s rank correlation test and Egger’s regression asymmetry test. A two-tailed *P* < 0.05 was statistically significant.

## Results

### Characteristics of studies

Eighty studies (42 cohort studies, 23 RCTs, and 15 case-control studies) encompassing 39,374,874 patients were ultimately included in our meta-analysis (Supplementary Figure 1). The 23 RCTs were strictly randomly assigned. In addition, the baseline data of the macrolides group and the non-macrolides group in other included studies were matched, and they were comparable as cardiovascular risk factors. Background use of macrolides in the experimental arm consisted of azithromycin (*n* = 46), clarithromycin (*n* = 28), erythromycin (*n* = 12) and roxithromycin (*n* = 7). Most underlying diseases treated with macrolides were respiratory infections. The main characteristics of the study population are detailed in Supplementary Table 1. The Newcastle-Ottawa Quality Assessment Scale scores of 57 observational studies ranged from 5 to 9, with 43 scoring 7 or above. The methodological quality of RCTs was generally good, with 21 studies scoring 4 or higher on the Modified Jadad score assessment (Supplementary Tables 2-4).

### Association with CVD events

**FIGURE 1:**
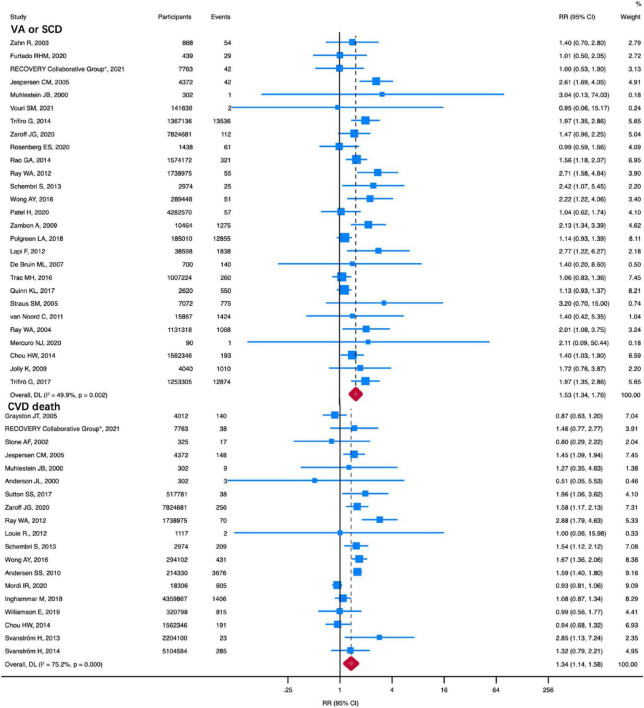
RR of VA or SCD and CVD death in patients using macrolides. Compared with no macrolides, macrolides were associated with higher VA or SCD (RR: 1.53, 95% CI: 1.34–1.76) and CVD death (RR: 1.34, 95% CI: 1.14–1.58). Squares represent mean values, with the size of the squares indicating weight and horizontal lines representing 95% CIs. The diamond represents the pooled mean with the points of the diamond representing 95% CIs. RR, relative risk; CI, confidence interval; VA or SCD, ventricular arrhythmia or sudden cardiac death; CVD, cardiovascular disease.

Compared with the non-macrolide treatment group, macrolides were associated with higher VA or SCD risk (RR: 1.53, 95% CI: 1.34–1.76, *I*^2^ = 49.9%) (Figure 1). A total of 27 studies, including 22,446,680 participants, were available for this analysis. We also performed a sensitivity analysis limiting the endpoint to SCD in eight studies, which showed an increased SCD risk associated with macrolides (RR: 1.81, 95% CI: 1.30-2.52, *I*^2^ = 66.3%) (Supplementary Figure 2). There was no visual asymmetry in the funnel plot (Supplementary Figure 3). Furthermore, Begg’s test and Egger’s regression asymmetry test indicated no evidence of publication bias (Begg test, *P* = 0.69; Egger test, *P* = 0.18).

Nineteen studies, including a total of 24,181,047 patients, reported CVD death. As shown in Figure 1, compared with the non-macrolide treatment group, macrolides were associated with higher CVD death (RR: 1.34, 95% CI: 1.14–1.58, *I*^2^ = 75.2%). A random-effects model was applied due to the high heterogeneity. No funnel plot asymmetry was observed (Supplementary Figure 3). Moreover, neither Egger’s test nor Begg’s test showed any publication bias (Begg test, *P* = 0.58; Egger test, *P* = 0.71).

The overall RR for MI comparing macrolides with no antibiotics for patients was 1.11 (95% CI: 1.02 to 1.20; *P* = 0.01) (Supplementary Figure 8), with moderate heterogeneity between studies (*I*^2^ = 74.5%, *P* < 0.001). Funnel plot distributions of MI revealed the absence of publication bias (Begg test, *P* = 0.77; Egger test, *P* = 0.77) (Supplementary Figure 3).

**FIGURE 2:**
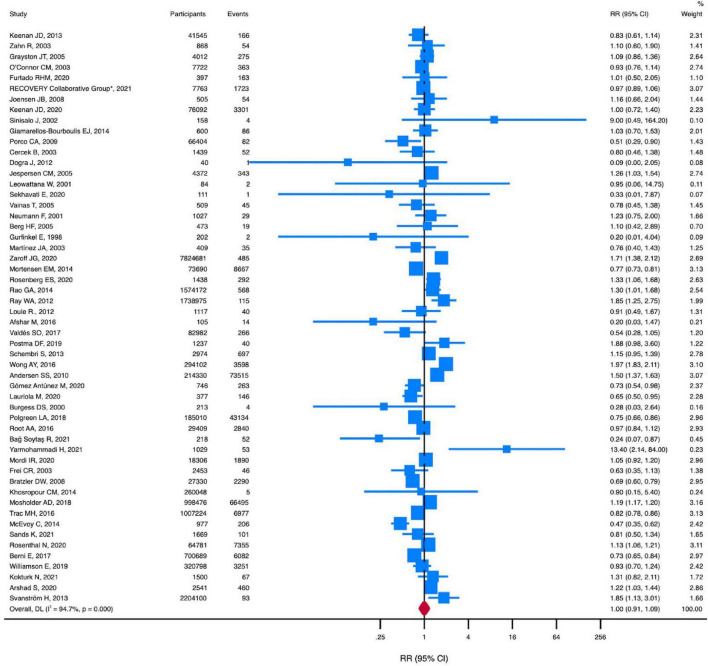
RR of all-cause death in patients using macrolides. Squares represent mean values, with the size of the squares indicating weight and horizontal lines representing 95% CIs. The diamond represents the pooled mean with the points of the diamond representing 95% CIs. RR, relative risk; CI, confidence interval.

Macrolides, compared with no macrolides, were not associated with a reduced risk of all-cause death (RR, 1.00 [95% CI, 0.91-1.10]; *P* = 0.99; *I*^2^ = 94.8%) (Figure 2). There was no evidence of publication bias for all-cause death (Begg test, *P* = 0.47; Egger test, *P* = 0.08) (Supplementary Figure 3).

### Individual drug and CVD risk

To confirm the strength of our study findings, we conducted a subgroup analysis evaluating the association between different types of macrolide antibiotics and cardiovascular risk (Table 1).

**TABLE 1:** Summary of Individual drug and timing of macrolides use subgroup analysis.

	VA or SCD		CVD death		MI		All-cause death	
**Type of macrolides**	**RR (95% CI)**	***p*-value**	**RR (95% CI)**	***p-*value**	**RR (95% CI)**	***p-*value**	**RR (95% CI)**	***p-*value**
Clarithromycin	1.52 (1.07-2.16)	0.02	1.22 (0.97-1.53)	0.10	1.24 (0.89-1.72)	0.20	1.14 (0.97-1.35)	0.11
Azithromycin	1.53 (1.19-1.97)	0.001	1.63 (1.17-2.27)	0.004	1.08 (1.02-1.15)	0.01	1.00 (0.88-1.13)	0.94
Erythromycin	1.47 (0.85-2.55)	0.17	-	-	0.98 (0.90-1.06)	0.57	-	-
Roxithromycin	1.40 (0.70-2.80)	0.34	1.04 (0.72-1.51)	0.84	1.01 (0.74-1.38)	0.94	1.15 (0.84-1.56)	0.39
**Timing of macrolides**								
Current	1.71 (1.48-1.99)	<0.001	1.54 (1.12-2.12)	0.01	1.66 (0.63-4.32)	0.30	1.27 (0.97-1.66)	0.09
Recent	1.25 (1.09-1.44)	0.001	1.14 (1.00-1.29)	0.06	1.19 (0.96-1.47)	0.12	0.96 (0.81-1.14)	0.66
Former	1.08 (0.93-1.24)	0.15	1.05 (0.84-1.30)	0.68	1.09 (1.02-1.15)	0.01	0.86 (0.72-1.02)	0.09

RR, relative risk; CI, confidence interval; VA or SCD, ventricular arrhythmia or sudden cardiac death; CVD, cardiovascular disease; MI, myocardial infarction; Current referred to the patient’s current use of macrolides, recent was defined as use of macrolides within one month, and former involved the use of macrolides within one year.

**FIGURE 3:**
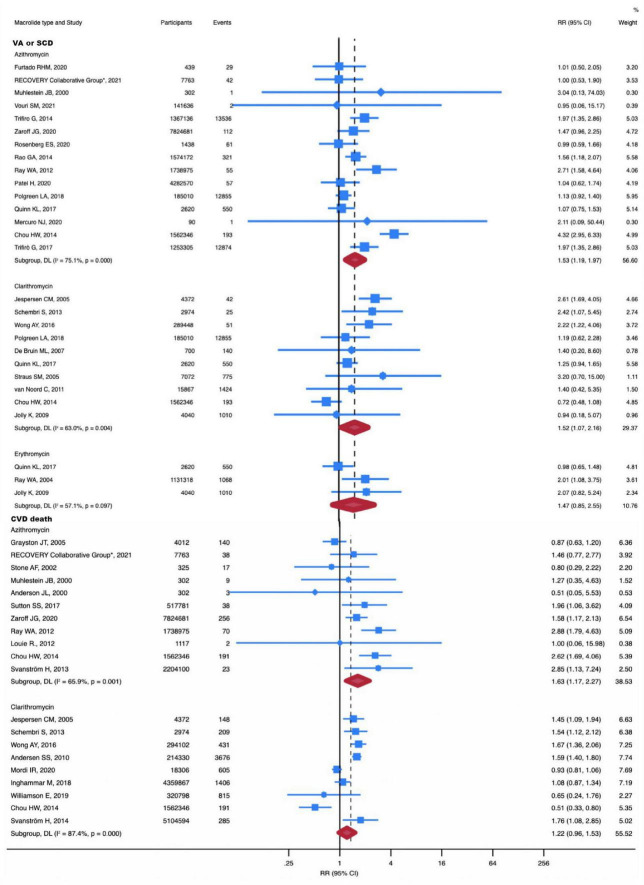
RR of VA or SCD and CVD death in individual drug subgroup analysis. The risk of VA or SCD was increased with azithromycin (RR, 1.63; 95% CI, 1.17-2.27) and clarithromycin (RR, 1.52; 95% CI, 1.07-2.16). The risk of CVD death was increased with azithromycin (RR, 1.63; 95% CI, 1.17-2.27). Squares represent mean values, with the size of the squares indicating weight and horizontal lines representing 95% CIs. The diamond represents the pooled mean with the points of the diamond representing 95% CIs. RR, relative risk; CI, confidence interval; VA or SCD, ventricular arrhythmia or sudden cardiac death; CVD, cardiovascular disease.

A total of 13 studies were included in the subgroup analysis of clarithromycin. No significant differences in MI, CVD, and all-cause death were found for clarithromycin use. Nonetheless, patients treated with clarithromycin had a higher risk of VA or SCD (RR, 1.52; 95% CI, 1.07-2.16; *P* = 0.02; *I*^2^ = 63.0%) (Figure 3).

With 28 studies included in the subgroup analysis of azithromycin, we concluded that azithromycin does not increase all-cause death. However, it should be noted that azithromycin significantly increased the risk of CVD death (RR, 1.63; 95% CI, 1.17-2.27; *P* = 0.004; *I*^2^ = 65.9%) (Figure 3) and VA or SCD (RR, 1.53; 95% CI, 1.19-1.97; *P* = 0.001; *I*^2^ = 0%) (Figure 3). Additionally, azithromycin increased the incidence of MI (RR, 1.08; 95% CI, 1.02-1.15; *P* = 0.014; *I*^2^ = 75.1%) (Supplementary Figure 4).

The number of studies for erythromycin was insufficient to perform meta-analyses for the outcomes of all-cause death and cardiovascular death. Three studies compared MI and VA or SCD in erythromycin versus no antibiotic treatment. Our subgroup analysis showed that erythromycin did not increase the risk of MI and VA or SCD.

Similarly, no significant differences were noted for roxithromycin in the incidence of MI (RR, 1.01; 95% CI, 0.74-1.38; *P* = 0.94; *I*^2^ = 0%) (Supplementary Figure 4) and all-cause death (RR, 1.14; 95% CI, 0.84-1.56; *P* = 0.39; *I*^2^ = 0%) (Supplementary Figure 5). One study reported the occurrence of VA or SCD with roxithromycin (RR, 1.40; 95% CI, 0.70-2.80; *P* = 0.34; *I*^2^ = 0%). Furthermore, another study reported the occurrence of CVD death with roxithromycin (RR, 1.04; 95% CI, 0.72-1.51; *P* = 0.84; *I*^2^ = 0%).

### Timing of macrolides use and CVD risk

Given that different timing of macrolide use was associated with different CVD risks, another subgroup analysis was conducted to investigate the association between timing of macrolide use and CVD risk (Table 1).

A total of 23 studies were included in the subgroup analysis of association between MI and different timing of macrolide use, including 14 studies in former subgroup, 6 studies in recent subgroup, and 3 studies in current subgroup. The subgroup analysis comprised 1,494,572 participants in total, of which 568,406 were in the former subgroup, 623,883 were in the recent subgroup, and 302,283 were in the current subgroup. Stratifying patients by the timing of macrolide use revealed an increased risk of MI for former use (RR, 1.09; 95% CI, 1.02-1.15; *P* = 0.01; *I*^2^ = 0%). Compared with non-antibiotic use, the current use of macrolides (RR, 1.66; 95% CI, 0.64-4.33; P = 0.30; *I*^2^ = 97.6%) and recent use of macrolides (RR, 1.19; 95% CI, 0.96-1.47; *P* = 0.12; *I*^2^ = 0%) did not increase the incidence of MI (Supplementary Figure 6).

**FIGURE 4:**
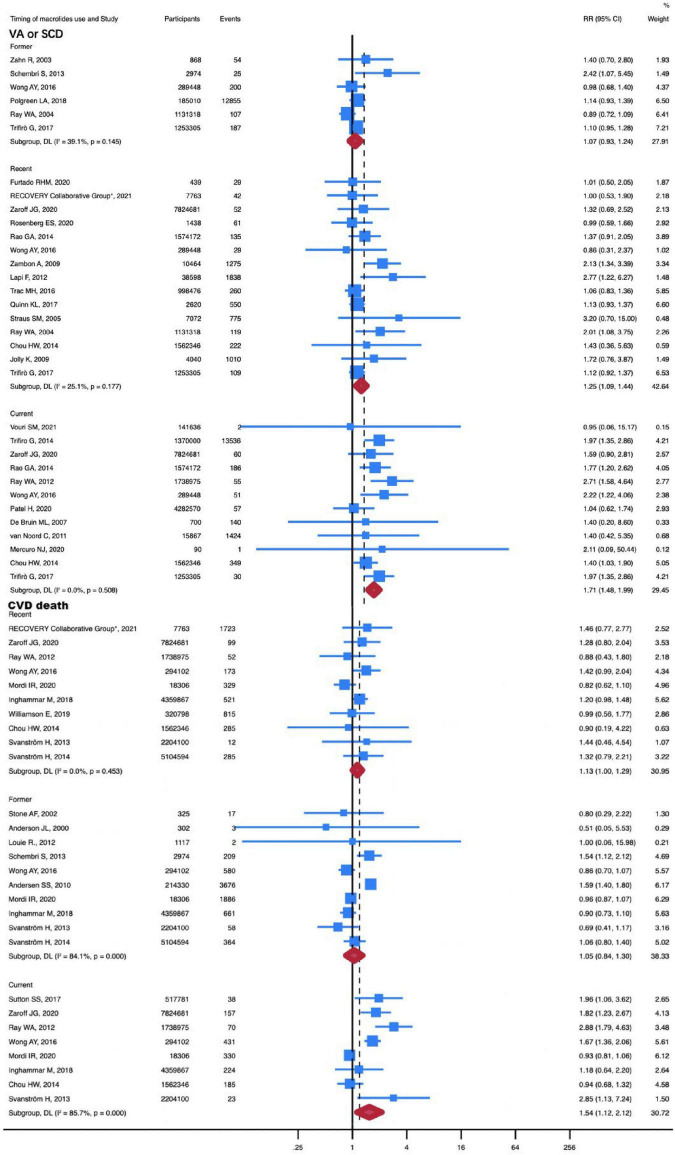
RR of VA or SCD and CVD death in timing of macrolides use subgroup analysis. The risk of VA or SCD was increased with recent (RR, 1.34; 95% CI, 1.09-1.65) and current (RR, 1.71; 95% CI, 1.48-1.99) use of macrolides. The risk of CVD death was increased with current (RR, 1.16; 95% CI, 1.02-1.32) use of macrolides. Squares represent mean values, with the size of the squares indicating weight and horizontal lines representing 95% CIs. The diamond represents the pooled mean with the points of the diamond representing 95% CIs. RR, relative risk; CI, confidence interval. VA or SCD, ventricular arrhythmia or sudden cardiac death; CVD, cardiovascular disease. Current referred to the patient’s current use of macrolides, recent was defined as use of macrolides within one month, and former involved the use of macrolides within one year.

The subgroup analysis of the association between MI and different timing of macrolide use comprised 33 studies in total, 6 of which were from the former subgroup, 15 from the recent subgroup, and 12 from the current subgroup. There were 37,622,893 participants in total, of which 2,862,923 were in the former subgroup, 14,706,180 in the recent subgroup, and 20,053,790 in the current subgroup. Notably, current use (RR, 1.71; 95% CI, 1.48-1.99; P < 0.001; *I*^2^ = 0%) and recent use (RR, 1.25; 95% CI, 1.09-1.44; P = 0.001; *I*^2^ = 25.1%) of macrolides increased the risk of VA or SCD. There was no increased risk of VA or SCD in patients with the former use of macrolides compared with those who had not taken antibiotics (RR, 1.08; 95% CI, 0.93-1.24; P = 0.15; *I*^2^ = 39.1%) (Figure 4).

50 studies with a total of 28,954,380 participants were used to investigate the association between different timing of macrolide use and all-cause death. The all-cause death was not increased in patients with current, recent, and former use of macrolides (Supplementary Figure 7). However, the risk of CVD death was elevated in patients with current use of macrolides (RR, 1.54; 95% CI, 1.12-2.12; *P* = 0.01; *I*^2^ = 85.7%) (Figure 4). In this subgroup, 28 studies with 54,155,707 participants were included. And the current subgroup contains 8 studies with a total of 1,8520,158 participants.

### Macrolides and CVD risk in patients with COVID-19

Compared with the non-macrolides group, macrolides were not associated with an increased risk of VA or SCD in patients with COVID-19 (RR: 1.01, 95% CI: 0.71-1.43, I**^2^** = 0%) (Supplementary Figure 9). A total of 4 studies, including 9,730 participants, were available for this analysis. Similarly, no significant differences in the incidence of all-cause death were observed in COVID-19 patients. (RR: 0.99, 95% CI: 0.85-1.15, *I***^2^** = 76.9%) (Supplementary Figure 10). A total of 11 studies with 81,070 participants were available for this analysis.

There was only one study on the association between macrolides use and CVD death in patients with COVID-19, and no study included the outcome of MI, therefore a meta-analysis on these two outcomes was not avaliable.

## Discussion

In this meta-analysis of 80 studies with a total of 39,374,874 participants and 134,629 CVD events, we demonstrated that the risk of CVD death, MI and VA or SCD was elevated with macrolide use. Subgroup analysis demonstrated that azithromycin was associated with an increased risk of 63% in CVD death, 8% in myocardial infarction and 53% in VA or SCD. Besides, patients prescribed clarithromycin had an increased risk of VA or SCD compared with no macrolide use. However, no CVD risk was associated with roxithromycin or erythromycin use. Analysis of the timing of macrolide use and CVD risk suggests that former macrolide use was associated with an increased risk of MI. Recent macrolide use was associated with an increased risk of VA or SCD. Moreover, current use of macrolides increased the incidence of VA or SCD and CVD death. Subgroup analysis also showed that macrolides did not lead to all-cause death and VA or SCD in patients with COVID-19.

A network meta-analysis reported no association between the use of macrolides and cardiovascular mortality or arrhythmia. Macrolides increase the risk of myocardial infarction, among which erythromycin and clarithromycin have been associated with a greater risk of myocardial infarction (10). Of note, previous meta-analyses have suggested that macrolide use can reduce all-cause death compared to no macrolide use (11, 12). Our previous studies suggested that clarithromycin can potentially increase the risk of all-cause death, cardiovascular death, and VA or SCD (3). In another study (13), clarithromycin was not associated with an increased risk of long-term all-cause death, cardiovascular death, or myocardial infarction. Moreover, it did not increase the incidence of arrhythmia, unlike the present study findings. In contrast with previous analyses, we included additional data from recently published studies, covering the largest study population and the four types of macrolides.

The mechanism of the association between macrolides and myocardial infarction remains unclear. It has been reported that clarithromycin may activate macrophages, leading to more vulnerable plaques through an inflammatory cascade that is more likely to trigger MI (14). It is widely acknowledged that clarithromycin is an inhibitor of cytochrome P450 3A4, while azithromycin does not inhibit cytochrome P450 3A4 (15). These pharmacokinetics makes clarithromycin at high risk of drug-drug interactions, such as impairing clopidogrel biotransformation and antiplatelet activity, which play an important role in the treatment and prevention of coronary heart disease (16, 17). However, we found that clarithromycin use did not increase the incidence of MI. Although azithromycin has been shown to reduce endothelial cell activation, our results suggest its use increases the incidence of MI (18). Interestingly, the current and recent use of macrolides does not cause MI, while former use can cause MI. This finding suggests that macrolide use may trigger atherosclerosis. It is well-established that atherosclerosis occurs under the influence of risk factors such as very low-density lipoprotein, but the key factor affecting the development of atherosclerosis is time (19). Although macrolides were also applied in the current and recent subgroups, the time for the onset and development of atherosclerosis was not more than 1 month, so it was difficult for patients to progress from the trigger of atherosclerosis to MI in a short time. However, patients in the former subgroup were given more time for atherosclerosis to develop than those in the other two subgroups, which may account for the higher risk of MI in the former subgroup.

The cardiotoxicity of macrolides is mainly manifested as prolonged QT interval and Torsades de pointes (TdP). The prolongation of phase 3 of the action potential, early afterdepolarization, and block of the cardiac delayed rectifier potassium current I(Kr) constitute the mechanism of VA or SCD in macrolides (20, 21). It has been established that the effect of macrolides on the blockade of the I(Kr) encoded by the human ether-a-go-go-related gene (HERG) can be ranked as follows: clarithromycin ≈ roxithromycin > erythromycin (22), explaining why clarithromycin tends to induce early after depolarizations and TdP. The absence of VA or SCD in patients formerly treated with macrolides suggests that the effect of the drugs on the I(Kr) may be time-limited. Azithromycin was once considered the safest macrolide because it minimally inhibits CYP3A4 and does not cause QT prolongation and TdP (22). However, the *in vitro* pharmacokinetic data of azithromycin may not reflect how it works *in vivo*. A previous study suggested that azithromycin may increase the risk of SCD (3). The combination of hydroxychloroquine and azithromycin effectively reduces the viral load in COVID-19 patients (23). However, when used independently, both drugs have been suggested to cause QT interval prolongation and TdP, which can induce SCD (24). In addition, it is now recommended to evaluate the QTc for COVID-19 patients who may require treatment with hydroxychloroquine and azithromycin (25).

In the present study, only azithromycin was significantly associated with an increased risk of CVD death among the four macrolides since azithromycin increases not only the risk of myocardial infarction but also the risk of VA or SCD. Moreover, clarithromycin did not increase CVD death rates, although it increased the incidence of VA or SCD.

None of the macrolides caused an increase in all-cause death. Even though azithromycin increased cardiovascular death, it did not cause an increase in all-cause death. Moreover, macrolide use reduced mortality in patients with severe sepsis caused by pneumonia (26). In addition, the anti-inflammatory and immunomodulatory properties of macrolides can reduce or mitigate the inflammatory response caused by pneumonia (27). Macrolides have been shown to regulate cytokines such as tumor necrosis factor α, interleukin 1, interleukin 6, interleukin 8 and interferon γ, which play an important role in host defense mechanisms in patients with pneumonia (27, 28). In light of the previous studies warning against the safety of azithromycin, physicians nowadays have to adjust the prescription of patients with excessive QT prolongation to decrease the risk of TdP (29–31). Careful prescription of azithromycin may result in fewer deaths from TdP and lower all-cause death rates.

Both the incidence of VA or SCD and the all-cause death rate were not elevated by macrolides in COVID-19 individuals. Previous study have shown that the COVID-19 infection may result in myocardial injury, which may subsequently lead to severe ventricular arrhythmias (32). However, our study reveals that macrolides can be prescribed and have good safety in COVID-19 patients. The administration of macrolide antibiotics may not directly cause ventricular arrhythmias, and there are other significant triggers, such as COVID-19 infection’s severe respiratory insufficiency, systemic inflammation, and proarrhythmic effects of COVID therapies (33, 34). Due to the limited number of studies at present, further clinical data is needed to determine if macrolides increase the risk of MI and CVD death in COVID-19.

Our study findings have important clinical implications. Due to differences in pharmacokinetic characteristics, drug-drug interactions and immunomodulatory effects among different macrolides, there are differences in the safety profile of different macrolides (35, 36). The cardiovascular risks associated with azithromycin and clarithromycin treatment in patients require caution. Notably, this study provides clinicians with directions for targeting different cardiovascular risks at different times in patients on macrolides. Electrocardiogram screening should be performed in high-risk patients when macrolides are prescribed. Indeed, azithromycin and clarithromycin should be avoided in patients with prolonged QT due to the risk of VA or SCD. Azithromycin should be carefully prescribed to avoid increasing the risk of MI if patients are taking drugs for secondary prevention of coronary heart disease. Our findings suggest that roxithromycin has a good safety profile in cardiovascular events and all-cause death. Based on the current literature, few studies have been conducted on erythromycin; thus, the risk of CVD death and all-cause death with erythromycin remains uncertain. Last but not least, this study provides new insights for clinicians on the safety of macrolide antibiotics in COVID-19.

Our study has several strengths. First, to our knowledge, the present study represents the largest and most comprehensive evaluation of the association between macrolides and CVD death risk. Besides, strict inclusion criteria were used, and no significant publication bias was found, indicating the robustness of the study findings. Moreover, we performed several subgroup and sensitivity analyses and observed no significant change in the magnitude of the direction of the pooled effect size for the association between macrolides and CVD death risk.

There were several limitations and shortcomings in this study. To include all data in the analysis, we pooled observational studies with RCTs. There were large heterogeneities in this meta-analysis, which may be due to different study types, selection of placebo or active comparators, and different baseline comorbidities. Therefore, we conducted subgroup analyses providing novel insights into the timing of macrolide use and the risk of the different macrolide antibiotics. Nonetheless, further studies are needed to validate our findings and implicated mechanisms.

## Conclusion

The findings of this meta-analysis indicate that macrolide use increases the risk of CVD death, VA or SCD, and myocardial infarction, although not increasing all-cause death. Both azithromycin and clarithromycin increase the incidence of VA or SCD, and azithromycin tends to increase the risk of CVD death and MI. Different CVD risks are increased with different timing of macrolide use.

## Data availability statement

The raw data supporting the conclusions of this article will be made available by the authors, without undue reservation.

## Author contributions

S-HW and Y-JC: full access to all of the data in the study and take responsibility for the integrity of the data and the accuracy of the data analysis and supervision. S-HW, Y-JC, YW, and W-TB: concept and design. S-HW, YW, and W-TB: acquisition, analysis, or interpretation of data. YW, W-TB, L-PQ, and JF: drafting of the manuscript. S-HW, Y-JC, YW, W-TB, X-JK, and C-CJ: critical revision of the manuscript for important intellectual content. YW, W-TB, X-MC, and F-JY: statistical analysis. All authors contributed to the article and approved the submitted version.
