# Supplementary material for: Administration of macrolide antibiotics increases cardiovascular risk
Source: Front Cardiovasc Med. 2023 Feb 23;10:1117254. doi: 10.3389/fcvm.2023.1117254 (PMC9996752; doi:10.3389/fcvm.2023.1117254)
Supplement: Supplementary file 1 [file Table_2.DOCX]

**Supplemental Appendix**

**Supplemental Table 1** Summary of Available Studies Included in the Present Meta-analysis

**Supplemental Table 2** Quality Assessment of Included Cohort Studies Using the Newcastle-Ottawa Scale

**Supplemental Table 3** Quality Assessment of Included Case-Control Studies Using the Newcastle-Ottawa Scale

**Supplemental Table 4** Quality Assessment of Included Randomized Controlled Studies Using the Modified Jadad Scores

**Supplemental Figure 1**. PRISMA Flow Diagram

**Supplemental Figure 2**. Sensitivity analysis of RR of SCD associated with macrolides.

**Supplemental Figure 3**. Funnel plots showing association of cardiovascular risk with macrolides.

**Supplemental Figure 4**. Individual drug subgroup analysis showing RR of MI.

**Supplemental Figure 5**. Individual drug subgroup analysis showing RR of all-cause death.

**Supplemental Figure 6**. Timing of macrolides use subgroup analysis showing RR of MI.

**Supplemental Figure 7**. Timing of macrolides use subgroup analysis showing RR of all-cause death.

**Supplemental Figure 8**. RR of MI.

**Supplemental Figure 9**. Macrolides and VA or SCD in patients with COVID-19.

**Supplemental Figure 10**. Macrolides and all-cause death in patients with COVID-19.

**Supplemental Table 1**Summary of Available Studies Included in the Present Meta-analysis

| **Study** | **Source of Participants** | **Country** | **Type of Study** | **Baseline Diseases** | **Type of Antibiotics** | **Doses of Antibiotics** | **Duration of Antibiotics** | **Outcomes** |
| --- | --- | --- | --- | --- | --- | --- | --- | --- |
| Mortensen et al. 2014 (1) | Veterans Administration Health Care System | USA | Population-based cohort | Pneumonia | Azithromycin | N/A | Short-term | Death from any cause, any cardiovascular events, myocardial infarction |
| Rao et al. 2014 (2) | VA Medical Centers | USA | Population-based cohort | Respiratory, gastrointestinal, genitourinary, wound infection | Azithromycin | N/A | 1-10 days | Death from any cause, ventricular arrhythmias or sudden cardiac death |
| Ray et al. 2012 (3) | Tennessee Medicaid program | USA | Population-based cohort | Respiratory, gastrointestinal, genitourinary, wound infection | Azithromycin | N/A | 1-10 days | Death from any cause, cardiovascular death, sudden cardiac death |
| Albert et al. 2011 (4) | Academic health  centers | USA | Randomized controlled trial | COPD | Azithromycin | 250 mg daily | 1 year | Death from any cause, cardiovascular death, any cardiovascular events |
| Keenan et al. 2013 (5) | TANA Trial | Ethiopia | Randomized controlled trial | Trachoma | Azithromycin | 1000 mg daily for adults, 20 mg/kg daily for children | 1 day | Death from any cause |
| Zahn et al. 2003 (6) | ANTIBIO Study | Germany | Randomized controlled trial | CHD | Roxithromycin | 300 mg daily | 6 weeks | Death from any cause, any cardiovascular events, cardiac arrest, myocardial infarction, stroke |
| Grayston et al. 2005 (7) | ACES Study | USA | Randomized controlled trial | CHD | Azithromycin | 600 mg daily | 1 year | Death from any cause, cardiovascular death, myocardial infarction, stroke |
| O'Connor et al. 2003 (8) | WIZARD Study | Multinational | Randomized controlled trial | CHD | Azithromycin | 600 mg daily for 3 days, 600 mg weekly for subsequent 11 weeks | 12 weeks | Death from any cause, any cardiovascular events, myocardial infarction |
| Schembri et al. 2013 (9) | EXODUS dataset, Edinburgh pneumonia study cohort | UK | Population-based cohort | COPD, pneumonia | Clarithromycin | N/A | Short-term | Death from any cause, cardiovascular death, sudden cardiac death; any cardiovascular event, myocardial infarction |
| Joensen et al. 2007 (10) | Viborg Hospital | Denmark | Randomized controlled trial | Peripheral artery disease | Roxithromycin | 300 mg daily | 28 days | Death from any cause |
| Andersen et al. 2010 (11) | Danish National Patient Register | Denmark | Population-based cohort | CHD, gastroduodenal ulcer | Clarithromycin | N/A | 7-10 days | Death from any cause, cardiovascular death |
| Poluzzi et al. 2009 (12) | FDA Adverse Event Reporting System | Multinational | Case-control study | Respiratory, gastrointestinal, genitourinary, wound infection | Azithromycin, clarithromycin | N/A | Short-term | Torsades de pointes |
| Zambon et al. 2009 (13) | National Health Service | Italy | Case-control study | Respiratory, gastrointestinal, genitourinary, wound infection | Azithromycin, clarithromycin, erythromycin, Roxithromycin | N/A | Short-term | Ventricular arrhythmias or sudden cardiac death |
| Gupta et al. 1997 (14) | St George’s Hospital | UK | Randomized controlled trial | CHD | Azithromycin | 500 mg daily | 3 days | Death from any cause, cardiovascular events |
| Stone et al. 2002 (15) | STAMINA Trial | UK | Randomized controlled trial | CHD | Azithromycin | 500 mg daily | 7 days | Cardiovascular death, myocardial infarction |
| Cercek et al. 2003 (16) | AZACS Trial | Multinational | Randomized controlled trial | CHD | Azithromycin | 500 mg daily for 1day, 250 mg daily for subsequent 4 days | 5 days | Death from any cause, myocardial infarction, any cardiovascular events |
| Giamarellos- Bourboulis et al. 2013 (17) | Hospitals in Greece | Greece | Randomized controlled trial | Gram-negative sepsis | Clarithromycin | 1000 mg daily | 4 days | Death from any cause |
| Sinisalo et al. 2002 (18) | CLARIFY Study | Finland | Randomized controlled trial | CHD | Clarithromycin | 500 mg daily | 3 months | Cardiovascular death, myocardial infarction, cardiovascular events, stroke |
| Winkel et al. 2011 (19) | CLARICOR Trial | Denmark | Randomized controlled trial | CHD | Clarithromycin | 500 mg daily | 14 days | Death from any cause, cardiovascular death, sudden cardiac death, myocardial infarction, any cardiovascular events, stroke |
| Khosropour et al. 2014 (20) | Oregon Public Health Division; Public Health-Seatle and King County | USA | Population-based cohort | Sexually transmitted disease | Azithromycin | N/A | Short-term | Death from any cause |
| McEvoy et al. 2014 (21) | Barnes Jewish Hospital | USA | Retrospective cohort | Pneumonia | Azithromycin, clarithromycin, | N/A | Short-term | Death from any cause |
| Straus et al. 2005 (22) | IPCI project | Netherlands | Case-control study | Respiratory, gastrointestinal, genitourinary, wound infection | Clarithromycin, erythromycin | N/A | Short-term | Sudden cardiac death |
| Ray et al. 2004 (23) | Tennessee Medicaid program | USA | Population-based cohort | Respiratory, gastrointestinal, genitourinary, wound infection | Erythromycin | N/A | Short-term | Sudden cardiac death |
| Dogra et al. 2012 (24) | Poly Clinic | India | Randomized controlled trial | CHD | Azithromycin | 500 mg daily for 5 days, 500 mg every 10 days for subsequent periods | 12 months | Death from any cause, any cardiovascular event |
| [Leowattana et al.](http://www.ncbi.nlm.nih.gov/pubmed?term=Leowattana%20W%5BAuthor%5D&cauthor=true&cauthor_uid=12002908) 2001 (25) | Siriraj Hospital | Thailand | Randomized controlled trial | CHD | Roxithromycin | 300 mg daily | 30 days | Cardiovascular events |
| [Muhlestein et al.](http://www.ncbi.nlm.nih.gov/pubmed?term=Muhlestein%20JB%5BAuthor%5D&cauthor=true&cauthor_uid=11023928) 2000 (26) | ACADEMIC Study | USA | Randomized controlled trial | CHD | Azithromycin | 500 mg daily for 3 days, 500 mg weekly for subsequent periods | 3 months | Cardiovascular death, sudden cardiac death, myocardial infarction, any cardiovascular events, stroke |
| Gurfinkel et al. 1997 (27) | ROXIS Study | Argentina | Randomized controlled trial | CHD | Roxithromycin | 300 mg daily | 30 days | Death from any cause, myocardial infarction |
| Vainas et al. 2005 (28) | SPACE Trial | Netherlands | Randomized controlled trial | Peripheral arterial disease | Azithromycin | 500 mg daily | 3 days | Death from any cause, myocardial infarction, stroke |
| Berg et al. 2005 (29) | Amphia Hospital | Netherlands | Randomized controlled trial | CHD | Clarithromycin | 500 mg daily | 16 days | Death from any cause, any cardiovascular events, myocardial infarction, stroke |
| Neumann et al. 2001 (30) | ISAR-3 | Germany | Randomized controlled trial | CHD | Roxithromycin | 300 mg daily | 28 days | Death from any cause, myocardial infarction |
| Svanstrom et al. 2013 (31) | Danish Civil Registration System | Denmark | Population-based cohort | Respiratory, gastrointestinal, genitourinary, wound infection | Azithromycin | N/A | 30 days | Death from any cause, cardiovascular death |
| Chou et al. 2014 (32) | Taiwan National Health Insurance database | China | Population-based cohort | Respiratory, gastrointestinal, genitourinary, wound infection | Azithromycin, clarithromycin | N/A | 7 days | Ventricular arrhythmias or sudden cardiac death, cardiovascular death |
| Svanstrom et al. 2014 (33) | Danish Civil Registration System | Denmark | Population-based cohort | Respiratory, gastrointestinal, genitourinary, wound infection | Clarithromycin, roxithromycin | N/A | 30 days | Cardiovascular death |
| Furtado et al. 2020 (34) | COALITION II | Brazil | Randomized controlled trial | Severe COVID-19 | Azithromycin | 500 mg | 10 days | Death from any cause, sudden cardiac death |
| Horby et al. 2021 (35) | RECOVERY Collaborative Group* | UK | Randomized controlled trial | SARS-CoV-2 infection | Azithromycin | 500 mg | 10 days or until disc | Death from any cause, cardiovascular death, stroke death, non-cardiovascular death, any arrhythmias |
| Keenan et al. 2020 (36) | MORDOR | Malawi, Niger,  and Tanzania | Randomized controlled trial | Meningitis, dysentery, malaria and pneumonia | Azithromycin | Approximately 20 mg per kg of bodyweight | N/A | Death from any cause |
| Sekhavati et al. 2020 (37) | Ziaeian Hospital in Tehran | Iran | Randomized controlled trial | COVID-19 | Azithromycin | Oral AZM 500 mg daily | 5 days | Death from any cause |
| Martı´nez et al. 2002 (38) | Hospital Clinic of  Barcelona | Spain | Retrospective  analysis | Bacteremic pneumonia | Erythromycin | N/A | N/A | Death from any cause |
| Vouri et al. 2020 (39) | IBM MarketScan Commercial Claims and Medicare Supplemental Databases | USA | Retrospective cohort study | Autoimmune diseases | Azithromycin or amoxicillin | N/A | N/A | Sudden cardiac arrest/Ventricular arrhythmias, any cardiovascular events |
| Bjerrum et al. 2005 (40) | The Hospital Discharge  Registry (HDR), the Odense University Pharmacoepidemiologic  Database (OPED) prescription module and the  OPED demographic module | Denmark | Population-based case-control study | Chlamydia  pneumoniae | Macrolides, tetracyclines, quinolones, penicillins | N/A | N/A | Myocardial infarction |
| Meier et al. 1999 (41) | The United Kingdom-based General Practice Database | UK | Population-based case-control  study | Myocardial infarction | Erythromycin, clarithromycin, azithromycin | 250mg | N/A | Myocardial infarction |
| Sutton et al. 2017 (42) | The South Carolina Medicaid claims and pharmacy databases | USA | Retrospective  observational analysis | Congestive heart  Failure, Chronic obstructive  pulmonary disease, Myocardial infarction, Diabetes | Azithromycin; clarithromycin | N/A | N/A | Cardiovascular death |
| Trifirò et al. 2014 (43) | 7 healthcare databases | Multinational | Nested case-control study | N/A | Azithromycin | N/A | N/A | Ventricular arrhythmia |
| Zaroff et al. 2020 (44) | 2 large, diverse, community-based integrated care delivery systems | USA | Retrospective cohort study | N/A | Azithromycin | 250 mg, 500 mg | N/A | Death from any cause, cardiovascular death, sudden cardiac death, non-cardiovascular death |
| Rosenberg et al. 2020 (45) | New York City metropolitan region | USA | Retrospective multicenter cohort study | COVID-19 | Azithromycin | N/A | N/A | Death from any cause, sudden cardiac death |
| Afshar et al. 2016 (46) | Patients admitted to medical  and surgical ICUs at the University of Colorado | USA | Retrospective cohort | Severe sepsis | Azithromycin | N/A | Within the first 4 days of ICU admission and for a minimum duration of 48 hours | Death from any cause |
| Valdés et al. 2016 (47) | The Pediatric Health Information System database | USA | Retrospective cohort study | Community-acquired pneumonia | Azithromycin | N/A | N/A | Death from any cause, sudden cardiac death |
| Postma et al. 2019 (48) | CAP-START | Netherlands | Cohort study | Community-acquired pneumonia | Azithromycin, clarithromycin, and erythromycin | N/A | N/A | Death from any cause, any cardiovascular event, heart failure, any arrhythmias |
| Wong et al. 2016 (49) | Public healthcare services in Hong Kong | China | Cohort study | Chronic obstructive pulmonary disease and community acquired pneumonia | Clarithromycin | N/A | N/A | Death from any cause, any cardiovascular event, non-cardiovascular death, myocardial infarction, stroke, ventricular arrhythmia |
| Antúnez et al. 2020 (50) | SEMI-COVID registry | Spain | Retrospective cohort study | COVID-19 | Macrolides | N/A | N/A | Death from any cause |
| Patel et al. 2016 (51) | The Truven Health Analytics MarketScan database | USA | Cohort study | Acute bacterial infection | Azithromycin | N/A | At least 30 days | Sudden cardiac death, any cardiovascular event, any cardiovascular events, ventricular arrhythmia |
| Lauriola et al. 2020 (52) | Policlinico of Monza | Italy | Retrospective single-center cohort study | COVID-19 | Azithromycin | 500 mg | 10 days | Death from any cause |
| Burgess et al. 2000 (53) | St. Luke's Baptist Hospital | USA | Cohort study | Community-Acquired Pneumonia | Macrolides | N/A | N/A | Death from any cause |
| Polgreen et al. 2018 (54) | The Chronic Condition Data  Warehouse | USA | Cohort study | Acute myocardial infarction | Azithromycin | N/A | N/A | Death from any cause, any cardiovascular event, myocardial infarction, atrial fibrillation, ventricular arrhythmia |
| Root et al. 2016 (55) | Clinical Practice Research Datalink | UK | Cohort study | Helicobacter pylori | Clarithromycin | N/A | N/A | Myocardial infarction, death from any cause, stroke, any arrhythmias, non-cardiovascular death |
| SOYTAŞ et al. 2021 (56) | İstanbul University-Cerrahpaşa, Cerrahpaşa Medical Faculty Hospital | Turkey | Retrospective study | COVID-19 | Azithromycin | N/A | N/A | Death from any cause |
| Lapi et al. 2012 (57) | The healthcare databases from the province of Quebec | Canada | Cohort study | Respiratory conditions | Macrolide | N/A | N/A | Ventricular arrhythmia |
| Yarmohammadi et al. 2021 (58) | Columbia University Medical Center | USA | Case-control study | COVID-19 | Azithromycin | An initial  dose of 500 mg for one day, followed by 250 mg daily  for 4 additional days. | 5days | Death from any cause |
| Mordi et al. 2020 (59) | The community in Tayside | UK | Observational cohort study | With and without type 2 diabetes | Clarithromycin | N/A | N/A | Death from any cause, any cardiovascular event, myocardial infarction, cardiovascular death, non-cardiovascular death |
| Frei et al. 2003 (60) | U.S. Community-Acquired Pneumonia Project | USA | Retrospective study | Community-acquired pneumonia | Macrolide | N/A | N/A | Death from any cause |
| Bruin et al. 2006 (61) | Academic Medical  Centre, Amsterdam | Netherlands | Case–control study | Patients experiencing circulatory arrest | Clarithromycin, erythromycin | N/A | N/A | Sudden cardiac death |
| Bratzler et al. 2008 (62) | The National Pneumonia Project | USA | Retrospective study | Pneumonia | Macrolide | N/A | N/A | Death from any cause |
| Jackson et al. 2008 (63) | Group Health Cooperative of Puget Sound | USA | Retrospective, population-based case-control study | Pneumoniae | Erythromycin | 2g | N/A | Myocardial infarction |
| Inghammar et al. 2017 (64) | Danish National Prescription Registry | Sweden | Cohort study | Pneumonia | Clarithromycin  Roxithromycin | N/A | N/A | Cardiovascular death, non-cardiovascular death |
| Mosholder et al. 2017 (65) | UK Clinical Practice Research Datalink | UK | Retrospective cohort study | Pneumoniae | Clarithromycin | N/A | N/A | Death from any cause, myocardial infarction, stroke |
| Trac et al. 2016 (66) | 8 databases | Canada | Retrospective cohort study | Pneumoniae | Azithromycin, clarithromycin, erythromycin | N/A | N/A | Death from any cause, ventricular arrhythmia |
| Quinn et al. 2017 (67) | CDSERN | Canada | Population-based, nested, case–control  study | N/A | Azithromycin, clarithromycin, erythromycin | N/A | N/A | Sudden cardiac death |
| Sands et al. 2020 (68) | Facilities affiliated with a large healthcare system | USA | Retrospective analysis | COVID-19 | Azithromycin | N/A | N/A | Death from any cause |
| Noord et al. 2009 (69) | The Integrated Primary  Care Information project | Netherlands | Case-control study | N/A | Clarithromycin, erythromycin | N/A | N/A | Sudden cardiac death |
| Jackson et al. 2000 (70) | Group Health Cooperative  of Puget Sound | USA | Case-control study | Chlamydia pneumoniae | Erythromycin | N/A | N/A | Myocardial infarction |
| Luchsinger et al. 2001 (71) | Protocare Sciences | USA | Cohort study | Pneumoniae | Macrolides | N/A | N/A | Myocardial infarction |
| Rosenthal et al. 2020 (72) | Premier Healthcare Database | USA | Cohort study | COVID-19 | Azithromycin | N/A | N/A | Death from any cause |
| Berni et al. 2017 (73) | The UK Clinical Practice Research Datalink and  the Hospital Episode Statistics | UK | Retrospective cohort study | Pneumoniae | Clarithromycin | N/A | N/A | Death from any cause, any arrhythmia, any cardiovascular events |
| Williamson et al. 2019 (74) | Clinical Practice Research Datalink | UK | Cohort study | Chronic rhinosinusitis | Clarithromycin | 250mg or 500mg | 1 week | Death from any cause, any arrhythmias, myocardial infarction, cardiovascular death, non-cardiovascular death, stroke |
| Mercuro et al. 2020 (75) | Academic tertiary care center in Boston | USA | Cohort study | COVID-19 | Azithromycin | N/A | N/A | Ventricular arrhythmia |
| Jolly et al. 2009 (76) | Community in the Midlands of England in Birmingham | UK | Case-control study | N/A | Clarithromycin | N/A | N/A | Sudden cardiac death |
| Kokturk et al. 2021 (77) | Zonguldak Bulent Ecevit University | Turkey | Cohort study | Pneumoniae | Azithromycin | N/A | N/A | Death from any cause |
| Arshad et al. 2020 (78) | The Henry Ford Health System | USA | Cohort study | COVID-19 | Azithromycin | 500 mg once daily on day 1  followed by 250 mg once daily for the next 4 days | 5days | Death from any cause |
| KARTER et al. 2003 (79) | Northern California Kaiser  Permanente Diabetes Registry | USA | Case-control study | Diabetes | Azithromycin | N/A | N/A | Myocardial infarction |
| Trifirò et al. 2017 (80) | A network of 7 population-based health care databases | 5 European countries | Case-control study | New antibiotic  users | Azithromycin | N/A | N/A | Ventricular arrhythmia |

ACADEMIC, Azithromycin in Coronary Artery Disease: Elimination of Myocardial Infection with Chlamydia; ACES, Azithromycin and Coronary events Study; ANTIBIO, antibiotic therapy after an acute myocardial infarction; AZACS, Azithromycin in Acute Coronary syndrome; CHD, coronary heart disease;

CLARICOR, Intervention with Clarithromycin in Patients with Stable Coronary Heart Disease; CLARIFY, Clarithromycin in Acute Coronary Syndrome Patients in Finland; Abbreviations: COPD, chronic obstructive pulmonary disease; EXODUS, Exacerbations of Obstructive Lung Disease managed in UK Secondary careCare;

IPCI, Integrated Primary Care Information; ISAR-3, intracoronary-stenting-and-antibiotic regimen Trial; ROXIS: Randomized Trial of Roxithromycin in Non-Q-Wave Coronary Syndromes; SPACE, Secondary Prevention of Atherosclerosis Through *Chlamydia* *pneumoniae* Eradication; STAMINA, South Thames Trial of Antibiotics in Myocardial Infarction and Unstable Angina; TANA, Trachoma Amelioration in Northern Amhara; VA, Veterans Administration; WIZARD, Zithromax for Atherosclerosis and its Related Disorders.

**Appendix References**

1. Mortensen EM, Halm EA, Pugh MJ, Copeland LA, Metersky M, Fine MJ, et al. Association of azithromycin with mortality and cardiovascular events among older patients hospitalized with pneumonia. Jama. 2014;311(21):2199-208.

2. Rao GA, Mann JR, Shoaibi A, Bennett CL, Nahhas G, Sutton SS, et al. Azithromycin and levofloxacin use and increased risk of cardiac arrhythmia and death. Ann Fam Med. 2014;12(2):121-7.

3. Ray WA, Murray KT, Hall K, Arbogast PG, Stein CM. Azithromycin and the risk of cardiovascular death. N Engl J Med. 2012;366(20):1881-90.

4. Louie R. Azithromycin and the risk of cardiovascular death. N Engl J Med. 2012;367(8):774; author reply 5.

5. Keenan JD, Emerson PM, Gaynor BD, Porco TC, Lietman TM. Adult mortality in a randomized trial of mass azithromycin for trachoma. JAMA Intern Med. 2013;173(9):821-3.

6. Zahn R, Schneider S, Frilling B, Seidl K, Tebbe U, Weber M, et al. Antibiotic therapy after acute myocardial infarction: a prospective randomized study. Circulation. 2003;107(9):1253-9.

7. Grayston JT, Kronmal RA, Jackson LA, Parisi AF, Muhlestein JB, Cohen JD, et al. Azithromycin for the secondary prevention of coronary events. N Engl J Med. 2005;352(16):1637-45.

8. O'Connor CM, Dunne MW, Pfeffer MA, Muhlestein JB, Yao L, Gupta S, et al. Azithromycin for the secondary prevention of coronary heart disease events: the WIZARD study: a randomized controlled trial. Jama. 2003;290(11):1459-66.

9. Schembri S, Williamson PA, Short PM, Singanayagam A, Akram A, Taylor J, et al. Cardiovascular events after clarithromycin use in lower respiratory tract infections: analysis of two prospective cohort studies. Bmj. 2013;346:f1235.

10. Joensen JB, Juul S, Henneberg E, Thomsen G, Ostergaard L, Lindholt JS. Can long-term antibiotic treatment prevent progression of peripheral arterial occlusive disease? A large, randomized, double-blinded, placebo-controlled trial. Atherosclerosis. 2008;196(2):937-42.

11. Andersen SS, Hansen ML, Norgaard ML, Folke F, Fosbøl EL, Abildstrøm SZ, et al. Clarithromycin use and risk of death in patients with ischemic heart disease. Cardiology. 2010;116(2):89-97.

12. Poluzzi E, Raschi E, Moretti U, De Ponti F. Drug-induced torsades de pointes: data mining of the public version of the FDA Adverse Event Reporting System (AERS). Pharmacoepidemiol Drug Saf. 2009;18(6):512-8.

13. Zambon A, Polo Friz H, Contiero P, Corrao G. Effect of macrolide and fluoroquinolone antibacterials on the risk of ventricular arrhythmia and cardiac arrest: an observational study in Italy using case-control, case-crossover and case-time-control designs. Drug Saf. 2009;32(2):159-67.

14. Gupta S, Leatham EW, Carrington D, Mendall MA, Kaski JC, Camm AJ. Elevated Chlamydia pneumoniae antibodies, cardiovascular events, and azithromycin in male survivors of myocardial infarction. Circulation. 1997;96(2):404-7.

15. Stone AF, Mendall MA, Kaski JC, Edger TM, Risley P, Poloniecki J, et al. Effect of treatment for Chlamydia pneumoniae and Helicobacter pylori on markers of inflammation and cardiac events in patients with acute coronary syndromes: South Thames Trial of Antibiotics in Myocardial Infarction and Unstable Angina (STAMINA). Circulation. 2002;106(10):1219-23.

16. Cercek B, Shah PK, Noc M, Zahger D, Zeymer U, Matetzky S, et al. Effect of short-term treatment with azithromycin on recurrent ischaemic events in patients with acute coronary syndrome in the Azithromycin in Acute Coronary Syndrome (AZACS) trial: a randomised controlled trial. Lancet. 2003;361(9360):809-13.

17. Giamarellos-Bourboulis EJ, Mylona V, Antonopoulou A, Tsangaris I, Koutelidakis I, Marioli A, et al. Effect of clarithromycin in patients with suspected Gram-negative sepsis: results of a randomized controlled trial. J Antimicrob Chemother. 2014;69(4):1111-8.

18. Sinisalo J, Mattila K, Valtonen V, Anttonen O, Juvonen J, Melin J, et al. Effect of 3 months of antimicrobial treatment with clarithromycin in acute non-q-wave coronary syndrome. Circulation. 2002;105(13):1555-60.

19. Winkel P, Hilden J, Fischer Hansen J, Hildebrandt P, Kastrup J, Kolmos HJ, et al. Excess sudden cardiac deaths after short-term clarithromycin administration in the CLARICOR trial: why is this so, and why are statins protective? Cardiology. 2011;118(1):63-7.

20. Khosropour CM, Capizzi JD, Schafer SD, Kent JB, Dombrowski JC, Golden MR. Lack of association between azithromycin and death from cardiovascular causes. N Engl J Med. 2014;370(20):1961-2.

21. McEvoy C, Micek ST, Reichley RM, Kan J, Hoban A, Hoffmann J, et al. Macrolides are associated with a better survival rate in patients hospitalized with community-acquired but not healthcare-associated pneumonia. Surg Infect (Larchmt). 2014;15(3):283-9.

22. Straus SM, Sturkenboom MC, Bleumink GS, Dieleman JP, van der Lei J, de Graeff PA, et al. Non-cardiac QTc-prolonging drugs and the risk of sudden cardiac death. Eur Heart J. 2005;26(19):2007-12.

23. Ray WA, Murray KT, Meredith S, Narasimhulu SS, Hall K, Stein CM. Oral erythromycin and the risk of sudden death from cardiac causes. N Engl J Med. 2004;351(11):1089-96.

24. Dogra J. Oral azithromycin in extended dosage schedule for chronic, subclinical Chlamydia pneumoniae infection causing coronary artery disease: a probable cure in sight? Results of a controlled preliminary trial. Int J Gen Med. 2012;5:505-9.

25. Leowattana W, Bhuripanyo K, Singhaviranon L, Akaniroj S, Mahanonda N, Samranthin M, et al. Roxithromycin in prevention of acute coronary syndrome associated with Chlamydia pneumoniae infection: a randomized placebo controlled trial. J Med Assoc Thai. 2001;84 Suppl 3:S669-75.

26. Muhlestein JB, Anderson JL, Carlquist JF, Salunkhe K, Horne BD, Pearson RR, et al. Randomized secondary prevention trial of azithromycin in patients with coronary artery disease: primary clinical results of the ACADEMIC study. Circulation. 2000;102(15):1755-60.

27. Gurfinkel E, Bozovich G, Daroca A, Beck E, Mautner B. Randomised trial of roxithromycin in non-Q-wave coronary syndromes: ROXIS Pilot Study. ROXIS Study Group. Lancet. 1997;350(9075):404-7.

28. Vainas T, Stassen FR, Schurink GW, Tordoir JH, Welten RJ, van den Akker LH, et al. Secondary prevention of atherosclerosis through chlamydia pneumoniae eradication (SPACE Trial): a randomised clinical trial in patients with peripheral arterial disease. Eur J Vasc Endovasc Surg. 2005;29(4):403-11.

29. Berg HF, Maraha B, Scheffer GJ, Quarles-van Ufford M, Vandenbroucke-Grauls CM, Peeters MF, et al. Treatment with clarithromycin prior to coronary artery bypass graft surgery does not prevent subsequent cardiac events. Clin Infect Dis. 2005;40(3):358-65.

30. Neumann F, Kastrati A, Miethke T, Pogatsa-Murray G, Mehilli J, Valina C, et al. Treatment of Chlamydia pneumoniae infection with roxithromycin and effect on neointima proliferation after coronary stent placement (ISAR-3): a randomised, double-blind, placebo-controlled trial. Lancet. 2001;357(9274):2085-9.

31. Svanström H, Pasternak B, Hviid A. Use of azithromycin and death from cardiovascular causes. N Engl J Med. 2013;368(18):1704-12.

32. Chou HW, Wang JL, Chang CH, Lai CL, Lai MS, Chan KA. Risks of cardiac arrhythmia and mortality among patients using new-generation macrolides, fluoroquinolones, and β-lactam/β-lactamase inhibitors: a Taiwanese nationwide study. Clin Infect Dis. 2015;60(4):566-77.

33. Svanström H, Pasternak B, Hviid A. Use of clarithromycin and roxithromycin and risk of cardiac death: cohort study. Bmj. 2014;349:g4930.

34. Furtado RHM, Berwanger O, Fonseca HA, Corrêa TD, Ferraz LR, Lapa MG, et al. Azithromycin in addition to standard of care versus standard of care alone in the treatment of patients admitted to the hospital with severe COVID-19 in Brazil (COALITION II): a randomised clinical trial. Lancet. 2020;396(10256):959-67.

35. Azithromycin in patients admitted to hospital with COVID-19 (RECOVERY): a randomised, controlled, open-label, platform trial. Lancet. 2021;397(10274):605-12.

36. Keenan JD, Arzika AM, Maliki R, Elh Adamou S, Ibrahim F, Kiemago M, et al. Cause-specific mortality of children younger than 5 years in communities receiving biannual mass azithromycin treatment in Niger: verbal autopsy results from a cluster-randomised controlled trial. Lancet Glob Health. 2020;8(2):e288-e95.

37. Sekhavati E, Jafari F, SeyedAlinaghi S, Jamalimoghadamsiahkali S, Sadr S, Tabarestani M, et al. Safety and effectiveness of azithromycin in patients with COVID-19: An open-label randomised trial. Int J Antimicrob Agents. 2020;56(4):106143.

38. Martínez JA, Horcajada JP, Almela M, Marco F, Soriano A, García E, et al. Addition of a macrolide to a beta-lactam-based empirical antibiotic regimen is associated with lower in-hospital mortality for patients with bacteremic pneumococcal pneumonia. Clin Infect Dis. 2003;36(4):389-95.

39. Vouri SM, Thai TN, Winterstein AG. An evaluation of co-use of chloroquine or hydroxychloroquine plus azithromycin on cardiac outcomes: A pharmacoepidemiological study to inform use during the COVID19 pandemic. Res Social Adm Pharm. 2021;17(1):2012-7.

40. Bjerrum L, Andersen M, Hallas J. Antibiotics active against Chlamydia do not reduce the risk of myocardial infarction. Eur J Clin Pharmacol. 2006;62(1):43-9.

41. Meier CR, Derby LE, Jick SS, Vasilakis C, Jick H. Antibiotics and risk of subsequent first-time acute myocardial infarction. Jama. 1999;281(5):427-31.

42. Sutton SS, Hyche S, Magagnoli J, Hardin JW. Appraisal of the cardiovascular risks of azithromycin: an observational analysis. J Comp Eff Res. 2017;6(6):509-17.

43. Trifiro G, Oteri A, de Ridder M, Rijnbeek P, Pecchioli S, Mazzaglia G, et al. Association of Azithromycin and Ventricular Arrhythmia: the ARITMO Project. Pharmacoepidemiology and Drug Safety. 2014;23:189-90.

44. Zaroff JG, Cheetham TC, Palmetto N, Almers L, Quesenberry C, Schneider J, et al. Association of Azithromycin Use With Cardiovascular Mortality. JAMA Netw Open. 2020;3(6):e208199.

45. Rosenberg ES, Dufort EM, Udo T, Wilberschied LA, Kumar J, Tesoriero J, et al. Association of Treatment With Hydroxychloroquine or Azithromycin With In-Hospital Mortality in Patients With COVID-19 in New York State. Jama. 2020;323(24):2493-502.

46. Afshar M, Foster CL, Layden JE, Burnham EL. Azithromycin use and outcomes in severe sepsis patients with and without pneumonia. J Crit Care. 2016;32:120-5.

47. Valdés SO, Kim JJ, Niu MC, de la Uz CM, Miyake CY, Moffett BS. Cardiac Arrest in Pediatric Patients Receiving Azithromycin. J Pediatr. 2017;182:311-4.e1.

48. Postma DF, Spitoni C, van Werkhoven CH, van Elden LJR, Oosterheert JJ, Bonten MJM. Cardiac events after macrolides or fluoroquinolones in patients hospitalized for community-acquired pneumonia: post-hoc analysis of a cluster-randomized trial. BMC Infect Dis. 2019;19(1):17.

49. Wong AY, Root A, Douglas IJ, Chui CS, Chan EW, Ghebremichael-Weldeselassie Y, et al. Cardiovascular outcomes associated with use of clarithromycin: population based study. Bmj. 2016;352:h6926.

50. Gómez Antúnez M, Muiño Míguez A, Bendala Estrada AD, Maestro de la Calle G, Monge Monge D, Boixeda R, et al. Clinical Characteristics and Prognosis of COPD Patients Hospitalized with SARS-CoV-2. Int J Chron Obstruct Pulmon Dis. 2020;15:3433-45.

51. Patel H, Calip GS, DiDomenico RJ, Schumock GT, Suda KJ, Lee TA. Comparison of Cardiac Events Associated With Azithromycin vs Amoxicillin. JAMA Netw Open. 2020;3(9):e2016864.

52. Lauriola M, Pani A, Ippoliti G, Mortara A, Milighetti S, Mazen M, et al. Effect of Combination Therapy of Hydroxychloroquine and Azithromycin on Mortality in Patients With COVID-19. Clin Transl Sci. 2020;13(6):1071-6.

53. Burgess DS, Lewis JS, 2nd. Effect of macrolides as part of initial empiric therapy on medical outcomes for hospitalized patients with community-acquired pneumonia. Clin Ther. 2000;22(7):872-8.

54. Polgreen LA, Riedle BN, Cavanaugh JE, Girotra S, London B, Schroeder MC, et al. Estimated Cardiac Risk Associated With Macrolides and Fluoroquinolones Decreases Substantially When Adjusting for Patient Characteristics and Comorbidities. J Am Heart Assoc. 2018;7(9).

55. Root AA, Wong AY, Ghebremichael-Weldeselassie Y, Smeeth L, Bhaskaran K, Evans SJ, et al. Evaluation of the risk of cardiovascular events with clarithromycin using both propensity score and self-controlled study designs. Br J Clin Pharmacol. 2016;82(2):512-21.

56. Bağ Soytaş R, Ünal D, Arman P, Suzan V, Emiroğlu Gedik T, Can G, et al. Factors affecting mortality in geriatric patients hospitalized with COVID-19. Turk J Med Sci. 2021;51(2):454-63.

57. Lapi F, Wilchesky M, Kezouh A, Benisty JI, Ernst P, Suissa S. Fluoroquinolones and the risk of serious arrhythmia: a population-based study. Clin Infect Dis. 2012;55(11):1457-65.

58. Yarmohammadi H, Morrow JP, Dizon J, Biviano A, Ehlert F, Saluja D, et al. Frequency of Atrial Arrhythmia in Hospitalized Patients With COVID-19. Am J Cardiol. 2021;147:52-7.

59. Mordi IR, Chan BK, Yanez ND, Palmer CNA, Lang CC, Chalmers JD. Genetic and pharmacological relationship between P-glycoprotein and increased cardiovascular risk associated with clarithromycin prescription: An epidemiological and genomic population-based cohort study in Scotland, UK. PLoS Med. 2020;17(11):e1003372.

60. Frei CR, Koeller JM, Burgess DS, Talbert RL, Johnsrud MT. Impact of atypical coverage for patients with community-acquired pneumonia managed on the medical ward: results from the United States Community-Acquired Pneumonia Project. Pharmacotherapy. 2003;23(9):1167-74.

61. De Bruin ML, Langendijk PN, Koopmans RP, Wilde AA, Leufkens HG, Hoes AW. In-hospital cardiac arrest is associated with use of non-antiarrhythmic QTc-prolonging drugs. Br J Clin Pharmacol. 2007;63(2):216-23.

62. Bratzler DW, Ma A, Nsa W. Initial antibiotic selection and patient outcomes: observations from the National Pneumonia Project. Clin Infect Dis. 2008;47 Suppl 3:S193-201.

63. Jackson LA, Smith NL, Heckbert SR, Grayston JT, Siscovick DS, Psaty BM. Lack of association between first myocardial infarction and past use of erythromycin, tetracycline, or doxycycline. Emerg Infect Dis. 1999;5(2):281-4.

64. Inghammar M, Nibell O, Pasternak B, Melbye M, Svanström H, Hviid A. Long-Term Risk of Cardiovascular Death With Use of Clarithromycin and Roxithromycin: A Nationwide Cohort Study. Am J Epidemiol. 2018;187(4):777-85.

65. Mosholder AD, Lee JY, Zhou EH, Kang EM, Ghosh M, Izem R, et al. Long-Term Risk of Acute Myocardial Infarction, Stroke, and Death With Outpatient Use of Clarithromycin: A Retrospective Cohort Study. Am J Epidemiol. 2018;187(4):786-92.

66. Trac MH, McArthur E, Jandoc R, Dixon SN, Nash DM, Hackam DG, et al. Macrolide antibiotics and the risk of ventricular arrhythmia in older adults. Cmaj. 2016;188(7):E120-e9.

67. Quinn KL, Macdonald EM, Gomes T, Mamdani MM, Huang A, Juurlink DN. Macrolides, Digoxin Toxicity and the Risk of Sudden Death: A Population-Based Study. Drug Saf. 2017;40(9):835-40.

68. Sands K, Wenzel R, McLean L, Korwek K, Roach J, Miller K, et al. No clinical benefit in mortality associated with hydroxychloroquine treatment in patients with COVID-19. Int J Infect Dis. 2021;104:34-40.

69. van Noord C, Sturkenboom MC, Straus SM, Witteman JC, Stricker BH. Non-cardiovascular drugs that inhibit hERG-encoded potassium channels and risk of sudden cardiac death. Heart. 2011;97(3):215-20.

70. Jackson LA, Smith NL, Heckbert SR, Grayston JT, Siscovick DS, Psaty BM. Past use of erythromycin, tetracycline, or doxycycline is not associated with risk of first myocardial infarction. J Infect Dis. 2000;181 Suppl 3:S563-5.

71. Luchsinger JA, Pablos-Méndez A, Knirsch C, Rabinowitz D, Shea S. Relation of antibiotic use to risk of myocardial infarction in the general population. Am J Cardiol. 2002;89(1):18-21.

72. Rosenthal N, Cao Z, Gundrum J, Sianis J, Safo S. Risk Factors Associated With In-Hospital Mortality in a US National Sample of Patients With COVID-19. JAMA Netw Open. 2020;3(12):e2029058.

73. Berni E, de Voogd H, Halcox JP, Butler CC, Bannister CA, Jenkins-Jones S, et al. Risk of cardiovascular events, arrhythmia and all-cause mortality associated with clarithromycin versus alternative antibiotics prescribed for respiratory tract infections: a retrospective cohort study. BMJ Open. 2017;7(1):e013398.

74. Williamson E, Denaxas S, Morris S, Clarke CS, Thomas M, Evans H, et al. Risk of mortality and cardiovascular events following macrolide prescription in chronic rhinosinusitis patients: a cohort study using linked primary care electronic health records. Rhinology. 2019;57(4):252-60.

75. Mercuro NJ, Yen CF, Shim DJ, Maher TR, McCoy CM, Zimetbaum PJ, et al. Risk of QT Interval Prolongation Associated With Use of Hydroxychloroquine With or Without Concomitant Azithromycin Among Hospitalized Patients Testing Positive for Coronavirus Disease 2019 (COVID-19). JAMA Cardiol. 2020;5(9):1036-41.

76. Jolly K, Gammage MD, Cheng KK, Bradburn P, Banting MV, Langman MJ. Sudden death in patients receiving drugs tending to prolong the QT interval. Br J Clin Pharmacol. 2009;68(5):743-51.

77. Kokturk N, Babayigit C, Kul S, Duru Cetinkaya P, Atis Nayci S, Argun Baris S, et al. The predictors of COVID-19 mortality in a nationwide cohort of Turkish patients. Respir Med. 2021;183:106433.

78. Arshad S, Kilgore P, Chaudhry ZS, Jacobsen G, Wang DD, Huitsing K, et al. Treatment with hydroxychloroquine, azithromycin, and combination in patients hospitalized with COVID-19. Int J Infect Dis. 2020;97:396-403.

79. Karter AJ, Thom DH, Liu J, Moffet HH, Ferrara A, Selby JV. Use of antibiotics is not associated with decreased risk of myocardial infarction among patients with diabetes. Diabetes Care. 2003;26(7):2100-6.

80. Trifirò G, de Ridder M, Sultana J, Oteri A, Rijnbeek P, Pecchioli S, et al. Use of azithromycin and risk of ventricular arrhythmia. Cmaj. 2017;189(15):E560-e8.

**Supplemental Table 2**Quality Assessment of Included Cohort Studies Using the Newcastle-Ottawa Scale

|  | **Selection** | | | | **Comparability** | | **Outcome** | | |  |
| --- | --- | --- | --- | --- | --- | --- | --- | --- | --- | --- |
| **Author** | **Representativeness of**  **Exposed**  **Cohort** | **Selection of Non-Exposed Cohort** | **Ascertainment**  **Of Exposure** | **Demonstration That Outcome**  **of Interest Was Not Present at Start of Study** | **Adjust for** **age** | **Adjust for other cardiovascular risk factors** | **Assessment of outcome** | **Follow-up**  **length** | **Loss to** **follow-up rate** | **Total Quality**  **Score** |
| Mortensen et al. 2014 | 1 | 1 | 1 | 0 | 1 | 1 | 1 | 1 | 0 | 7 |
| Rao GA,2014 | 1 | 1 | 1 | 1 | 1 | 1 | 1 | 1 | 0 | 8 |
| Ray WA,2012 | 1 | 1 | 1 | 1 | 1 | 1 | 1 | 1 | 0 | 8 |
| Schembri S, 2013 | 1 | 1 | 1 | 0 | 1 | 1 | 1 | 1 | 1 | 8 |
| Andersen SS, 2010 | 1 | 1 | 1 | 1 | 1 | 0 | 1 | 1 | 0 | 7 |
| Khosropour CM, 2014 | 1 | 1 | 1 | 1 | 0 | 0 | 1 | 1 | 1 | 8 |
| McEvoy C, 2014 | 0 | 1 | 1 | 1 | 0 | 0 | 1 | 1 | 0 | 5 |
| Ray WA, 2004 | 1 | 1 | 1 | 1 | 1 | 0 | 1 | 1 | 0 | 7 |
| Svanstrom H, 2013 | 1 | 1 | 1 | 1 | 1 | 1 | 1 | 1 | 1 | 9 |
| Svanstrom H, 2014 | 1 | 1 | 1 | 1 | 1 | 1 | 1 | 1 | 1 | 9 |
| Chou HW, 2014 | 1 | 1 | 1 | 1 | 1 | 1 | 1 | 1 | 0 | 8 |
| Martı´nez et al. 2002 | 1 | 1 | 1 | 1 | 0 | 1 | 1 | 1 | 0 | 7 |
| Vouri et al. 2020 | 1 | 1 | 1 | 0 | 0 | 1 | 1 | 0 | 0 | 5 |
| Sutton et al. 2017 | 1 | 1 | 1 | 1 | 1 | 1 | 1 | 1 | 0 | 8 |
| Zaroff et al. 2020 | 1 | 1 | 1 | 1 | 1 | 1 | 1 | 0 | 0 | 7 |
| Rosenberg et al. 2020 | 1 | 1 | 1 | 1 | 1 | 1 | 1 | 1 | 1 | 9 |
| Afshar et al. 2016 | 1 | 1 | 1 | 0 | 1 | 1 | 1 | 0 | 0 | 7 |
| Valdés et al. 2016 | 1 | 1 | 1 | 0 | 1 | 1 | 1 | 1 | 0 | 7 |
| Postma et al. 2019 | 1 | 1 | 1 | 1 | 0 | 1 | 1 | 0 | 0 | 6 |
| Wong et al. 2016 | 1 | 1 | 1 | 1 | 1 | 1 | 1 | 0 | 1 | 8 |
| Antúnez et al. 2020 | 1 | 1 | 1 | 1 | 1 | 1 | 1 | 0 | 1 | 8 |
| Patel et al. 2016 | 1 | 1 | 1 | 1 | 1 | 1 | 1 | 0 | 1 | 8 |
| Lauriola et al. 2020 | 1 | 1 | 1 | 1 | 1 | 1 | 1 | 0 | 1 | 8 |
| Burgess et al. 2000 | 1 | 1 | 1 | 1 | 1 | 1 | 1 | 1 | 0 | 8 |
| Polgreen et al. 2018 | 1 | 1 | 1 | 1 | 1 | 1 | 1 | 0 | 1 | 8 |
| Root et al. 2016 | 1 | 1 | 1 | 1 | 1 | 1 | 1 | 1 | 1 | 9 |
| SOYTAŞ et al. 2021 | 1 | 1 | 1 | 0 | 0 | 0 | 1 | 0 | 1 | 5 |
| Lapi et al. 2012 | 1 | 1 | 1 | 1 | 1 | 1 | 1 | 1 | 1 | 9 |
| Mordi et al. 2020 | 1 | 1 | 1 | 1 | 1 | 1 | 1 | 0 | 1 | 8 |
| Frei et al. 2003 | 1 | 1 | 1 | 0 | 1 | 1 | 1 | 0 | 1 | 7 |
| Bratzler et al. 2008 | 1 | 1 | 1 | 0 | 1 | 1 | 1 | 0 | 0 | 6 |
| Inghammar et al. 2017 | 1 | 1 | 1 | 0 | 1 | 1 | 1 | 0 | 0 | 6 |
| Mosholder et al. 2017 | 1 | 1 | 1 | 0 | 1 | 1 | 1 | 1 | 1 | 8 |
| Trac et al. 2016 | 1 | 1 | 1 | 0 | 1 | 1 | 1 | 0 | 1 | 7 |
| Sands et al. 2020 | 1 | 1 | 1 | 0 | 1 | 1 | 1 | 0 | 1 | 7 |
| Luchsinger et al. 2001 | 1 | 1 | 1 | 0 | 1 | 1 | 1 | 0 | 1 | 7 |
| Rosenthal et al. 2020 | 1 | 1 | 1 | 0 | 1 | 1 | 1 | 0 | 1 | 7 |
| Berni et al. 2017 | 1 | 1 | 1 | 1 | 1 | 1 | 1 | 1 | 1 | 9 |
| Williamson et al. 2019 | 1 | 1 | 1 | 1 | 1 | 1 | 1 | 1 | 1 | 9 |
| Mercuro et al. 2020 | 1 | 1 | 1 | 1 | 1 | 1 | 1 | 0 | 1 | 8 |
| Kokturk et al. 2021 | 1 | 1 | 1 | 0 | 1 | 1 | 1 | 0 | 1 | 7 |
| Arshad et al. 2020 | 1 | 1 | 1 | 1 | 1 | 1 | 1 | 0 | 1 | 8 |

The quality of included studies was assessed by the Newcastle Ottawa scale. A study can be awarded a maximum of one star for each

numbered item within the Selection and Outcome categories and a maximum of two stars for Comparability.

**Selection**: 1) Representativeness of exposed cohort: 1, study population truly or somewhat representative of a community/ population

based study; 0, study population was sampled from a special population, that is, population from a company, hospital patients, data from

the health insurance company or health examination organization, nurses.

2) Selection of non-exposed cohort: 1, drawn from the same community as the exposed cohort.

3) Ascertainment of exposure: 1, Validation of macrolides use with secure medical record; 0, no specific macrolides use validation method.

4) Demonstration that outcome was not present at start of study: 1, exclusion of participants with a history of severe ventricular arrhythmia or sudden cardiac arrest at the beginning of the study.

**Comparability**: 1) 1, whether a study adjusted for age deliberately; 1, whether a study adjusted for other cardiovascular risk factors.

**Outcome**: 1) Assessment of outcome: 1, cardiovascular events were confirmed by medical records or record linkage; 0, self-reported.

2) Was follow-up long enough for outcomes to occur: 1, duration of follow-up >= 5 year; 0, if duration of follow-up < 5 year.

3) Loss to follow-up rate: 1, complete follow-up or loss to follow up rate <=20 %; 0, follow-up rate < 80% or no description of those

lost.

**Supplemental Table 3**Quality Assessment of Included Case-Control Studies Using the Newcastle-Ottawa Scale

|  | **Selection** | | | | **Comparability** | | **Outcome** | | |  |
| --- | --- | --- | --- | --- | --- | --- | --- | --- | --- | --- |
| **Author** | **Adequacy of case definition** | **Representativeness of the cases** | **Selection of Controls** | **Definition of Controls** | **Adjust for Age** | **Adjust for Other Cardiovascular Risk Factors** | **Assessment of Outcome** | **Same Method of Ascertainment for Cases and Controls** | **Non-Response Rate** | **Total Quality**  **Score** |
| Poluzzi E, 2009 | 1 | 1 | 1 | 0 | 0 | 1 | 1 | 1 | 0 | 6 |
| Zambon A, 2009 | 1 | 1 | 1 | 0 | 0 | 1 | 1 | 1 | 0 | 6 |
| Straus SM, 2005 | 1 | 1 | 1 | 1 | 1 | 1 | 1 | 1 | 0 | 8 |
| Bjerrum et al. 2005 | 1 | 1 | 1 | 0 | 1 | 1 | 1 | 1 | 1 | 8 |
| Meier et al. 1999 | 1 | 1 | 0 | 1 | 1 | 1 | 1 | 1 | 0 | 7 |
| Trifirò et al. 2014 | 1 | 1 | 0 | 0 | 0 | 1 | 1 | 1 | 0 | 5 |
| Yarmohammadi et al. 2021 | 1 | 1 | 0 | 0 | 1 | 1 | 1 | 1 | 0 | 6 |
| Bruin et al. 2006 | 1 | 1 | 0 | 1 | 1 | 1 | 1 | 1 | 0 | 7 |
| Jackson et al. 2008 | 1 | 1 | 0 | 0 | 1 | 1 | 1 | 1 | 0 | 6 |
| Quinn et al. 2017 | 1 | 1 | 0 | 0 | 0 | 1 | 1 | 1 | 0 | 5 |
| Noord et al. 2009 | 1 | 1 | 0 | 0 | 1 | 1 | 1 | 1 | 0 | 6 |
| Jackson et al. 2000 | 1 | 1 | 0 | 0 | 1 | 1 | 1 | 1 | 0 | 6 |
| Jolly et al. 2009 | 1 | 1 | 1 | 1 | 0 | 1 | 1 | 1 | 0 | 7 |
| KARTER et al. 2003 | 1 | 1 | 0 | 0 | 1 | 1 | 1 | 1 | 1 | 7 |
| Trifirò et al. 2017 | 1 | 1 | 0 | 0 | 1 | 1 | 1 | 1 | 0 | 7 |

The quality of included studies was assessed by the Newcastle Ottawa scale. A study can be awarded a maximum of one star for each

numbered item within the Selection and Outcome categories and a maximum of two stars for Comparability.

**Selection**: 1) Adequacy of case definition: 1, cardiovascular events were confirmed by medical records or record linkage; 0, self-reported.

2) Representativeness of the cases: 1, consecutive or obviously representative series of cases; 0, potential for selection biases or not stated.

3) Selection of Controls: 1, community controls; 0, hospital controls or no description.

4) Definition of Controls: 1, no history of cardiovascular events; 0, no description of source.

**Comparability**: 1) 1, whether a study adjusted for age deliberately; 1, whether a study adjusted for other cardiovascular risk factors.

**Outcome**: 1) Assessment of outcome: 1, cardiovascular events were confirmed by medical records or record linkage; 0, self-reported.

2) Same method of ascertainment for cases and controls: 1, yes; 0, no.

3) Non-Response rate: 1, same rate for both groups; 0, non respondents described rate different and no designation.

**Supplemental Table 4**Quality Assessment of Included Randomized Controlled Studies Using the Modified Jadad Scores

| **Author** | **Randomization** | **Concealment of**  **Allocation** | **Double Blinding** | **Withdrawals and**  **Dropouts** | **Total** |
| --- | --- | --- | --- | --- | --- |
| Albert RK, 2011 | 1 | 1 | 2 | 1 | 5 |
| Keenan JD, 2013 | 2 | 2 | 0 | 1 | 5 |
| Zahn R, 2002 | 2 | 2 | 2 | 1 | 7 |
| Grayston JT, 2005 | 1 | 1 | 2 | 1 | 5 |
| O'Connor CM, 2003 | 2 | 2 | 2 | 1 | 7 |
| Joensen JB, 2007 | 1 | 1 | 2 | 1 | 5 |
| Gupta S, 1997 | 1 | 1 | 1 | 1 | 4 |
| Stone AF, 2002 | 1 | 1 | 2 | 1 | 5 |
| Cercek B, 2003 | 1 | 2 | 2 | 1 | 6 |
| [Giamarellos-Bourboulis EJ](http://www.ncbi.nlm.nih.gov/pubmed?term=Giamarellos-Bourboulis%20EJ%5BAuthor%5D&cauthor=true&cauthor_uid=24292991), 2013 | 2 | 2 | 2 | 1 | 7 |
| Sinisalo J, 2002 | 2 | 2 | 2 | 1 | 7 |
| Winkel P, 2011 | 2 | 1 | 1 | 1 | 5 |
| Dogra J, 2012 | 0 | 0 | 0 | 1 | 1 |
| [Leowattana W](http://www.ncbi.nlm.nih.gov/pubmed?term=Leowattana%20W%5BAuthor%5D&cauthor=true&cauthor_uid=12002908), 2001 | 1 | 1 | 1 | 1 | 4 |
| [Muhlestein JB](http://www.ncbi.nlm.nih.gov/pubmed?term=Muhlestein%20JB%5BAuthor%5D&cauthor=true&cauthor_uid=11023928), 2000 | 2 | 2 | 1 | 1 | 6 |
| Gurfinkel E, 1997 | 2 | 2 | 2 | 1 | 7 |
| Vainas T, 2005 | 2 | 2 | 1 | 1 | 7 |
| Berg HF, 2005 | 2 | 2 | 2 | 1 | 7 |
| Neumann F, 2001 | 2 | 2 | 2 | 1 | 7 |
| Furtado et al. 2020 | 2 | 2 | 2 | 1 | 7 |
| Horby et al. 2021 | 2 | 2 | 2 | 1 | 7 |
| Keenan et al. 2020 | 2 | 2 | 2 | 1 | 7 |
| Sekhavati et al. 2020 | 1 | 1 | 0 | 1 | 3 |

**Randomization:** 0, not randomized or inappropriate method of randomization; 1, the study was described as randomized; 2, the method of randomization was described and it was appropriate.

**Concealment of** **allocation:** 0, not describe the method of allocation concealment; 1, the study was described as using allocation concealment method; 2, the method of allocation concealment was described appropriately.

**Double** **blinding:** 0, no blind or inappropriate method of blinding; 1, the study was described as double blind; 2, the method of double blinding was described and it was appropriate.

**Withdrawals and dropouts:** 0, not describe the follow-up; 1, a description of withdrawals and dropouts.


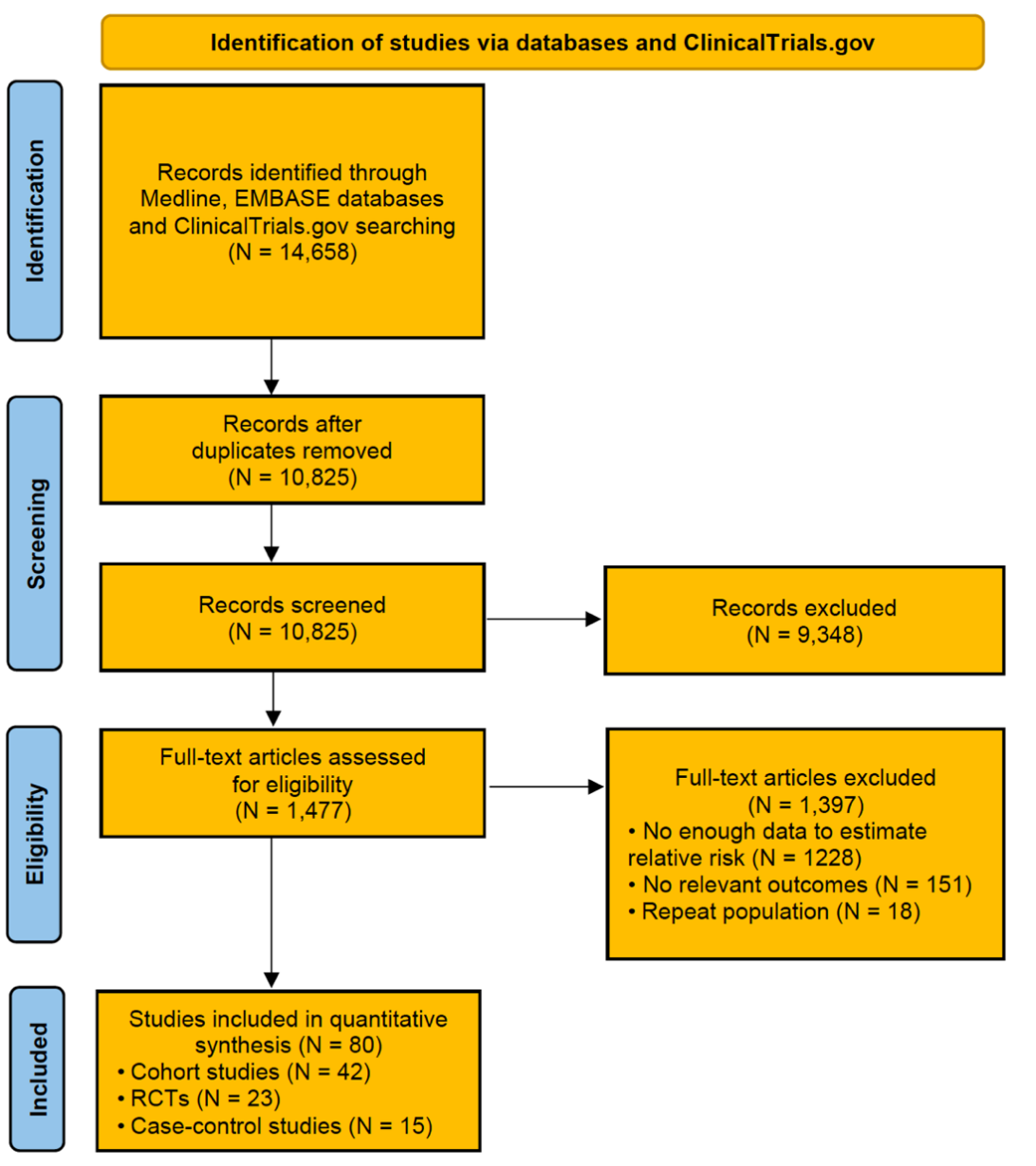


**Supplemental Figure 1**. PRISMA Flow Diagram. Medline, EMBASE databases and ClinicalTrials.gov were systematically searched for studies reporting on cardiovascular risk in patients treated with macrolides. A total of 80 studies were selected for meta-analysis. PRISMA = Preferred Reporting Items for Systematic Reviews and Meta-Analyses.


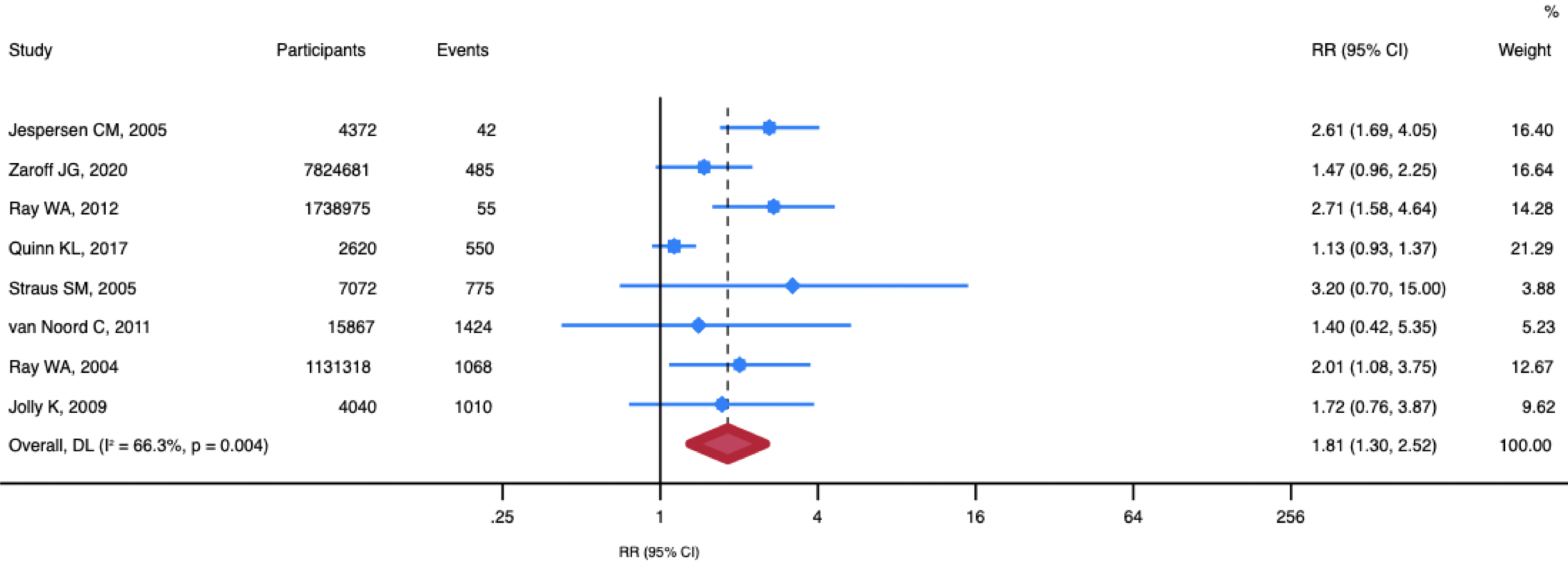


**Supplemental Figure 2**. Sensitivity analysis of RR of SCD associated with macrolides. **Squares** represent mean values, with the size of the squares indicating weight and **horizontal lines** representing 95% CIs. The **diamond** represents the pooled mean with the points of the diamond representing 95% CIs. **RR** = relative risk; **CI** = confidence interval; **SCD** = sudden cardiac death.


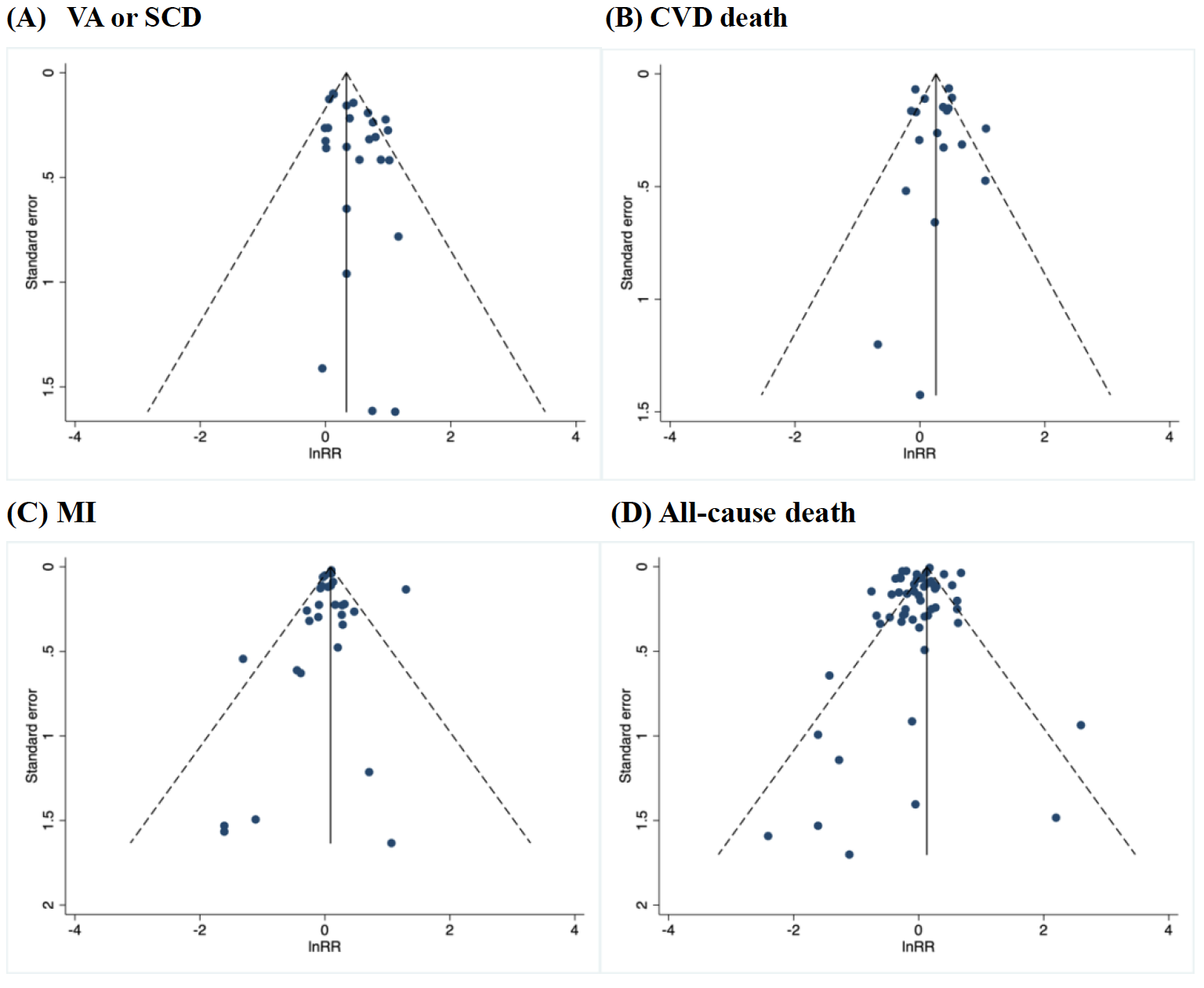


**Supplemental Figure 3**. Funnel plots showing association of cardiovascular risk with macrolides. **VA or SCD** = ventricular arrhythmia or sudden cardiac death; **CVD** = cardiovascular disease; **MI** = myocardial infarction;


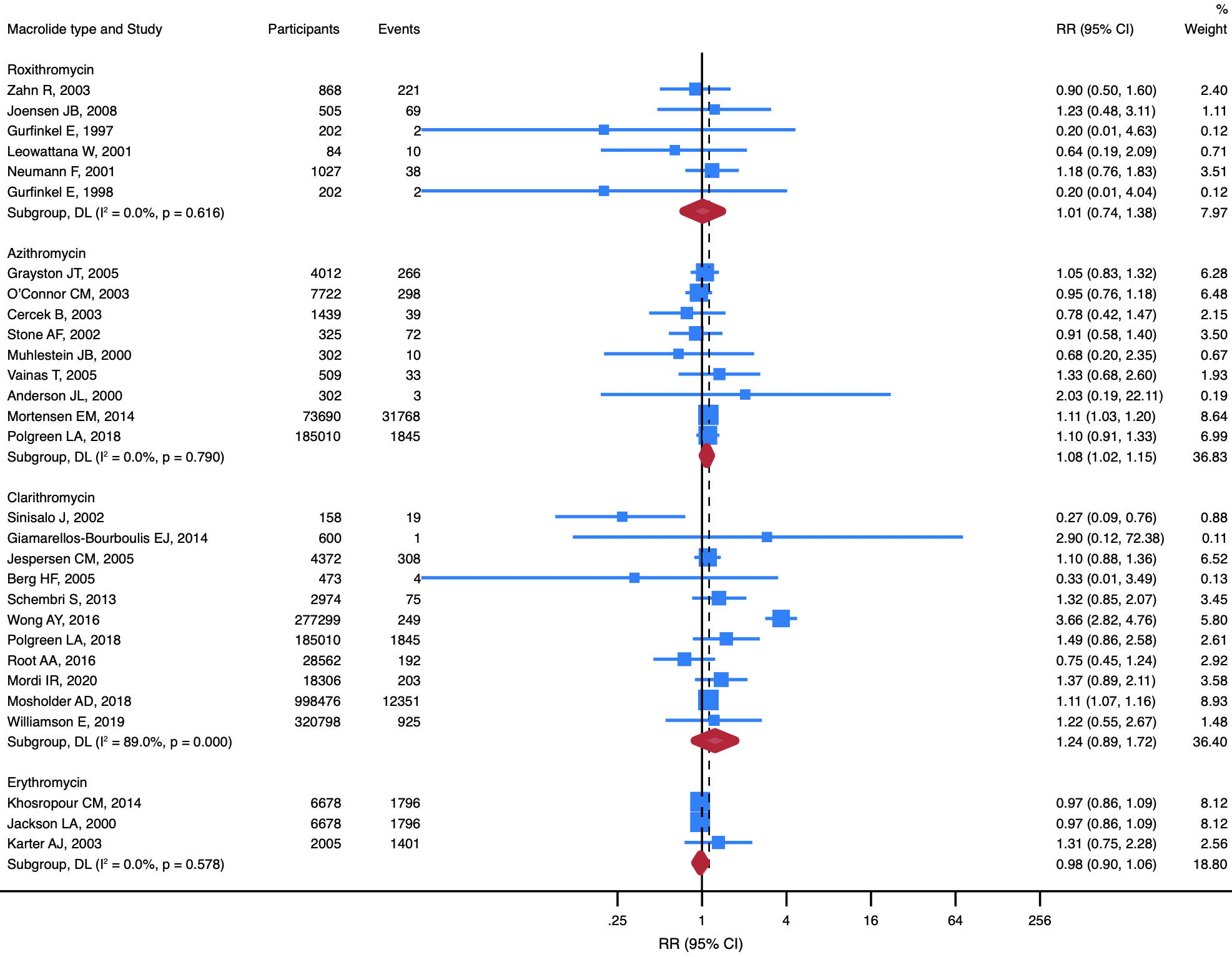


**Supplemental Figure 4**. Individual drug subgroup analysis showing RR of MI. **Squares** represent mean values, with the size of the squares indicating weight and **horizontal lines** representing 95% CIs. The **diamond** represents the pooled mean with the points of the diamond representing 95% CIs. **RR** = relative risk; **CI** = confidence interval; **MI** = myocardial infarction.


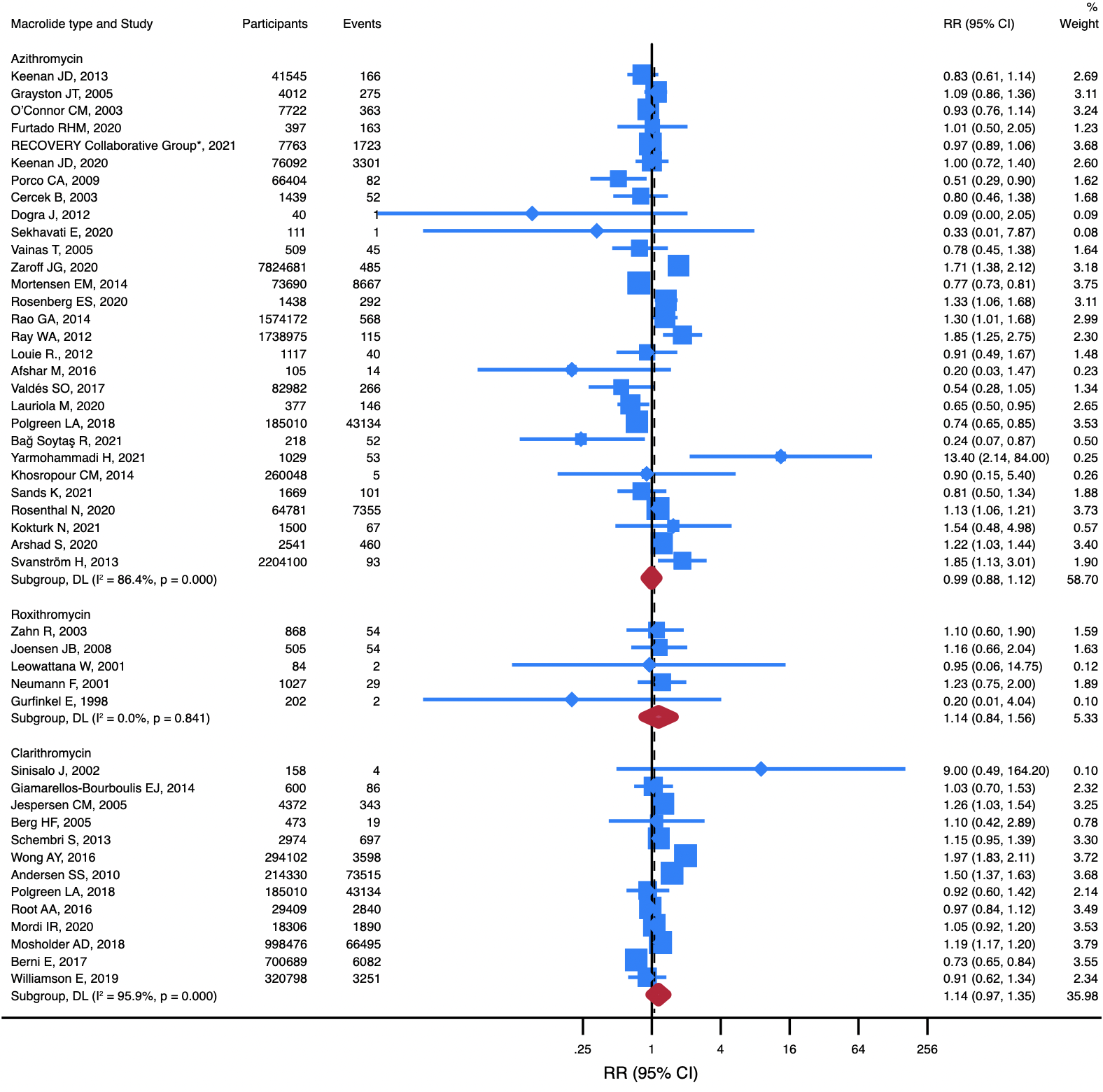


**Supplemental Figure 5**. Individual drug subgroup analysis showing RR of all-cause death. **Squares** represent mean values, with the size of the squares indicating weight and **horizontal lines** representing 95% CIs. The **diamond** represents the pooled mean with the points of the diamond representing 95% CIs. **RR** = relative risk; **CI** = confidence interval.


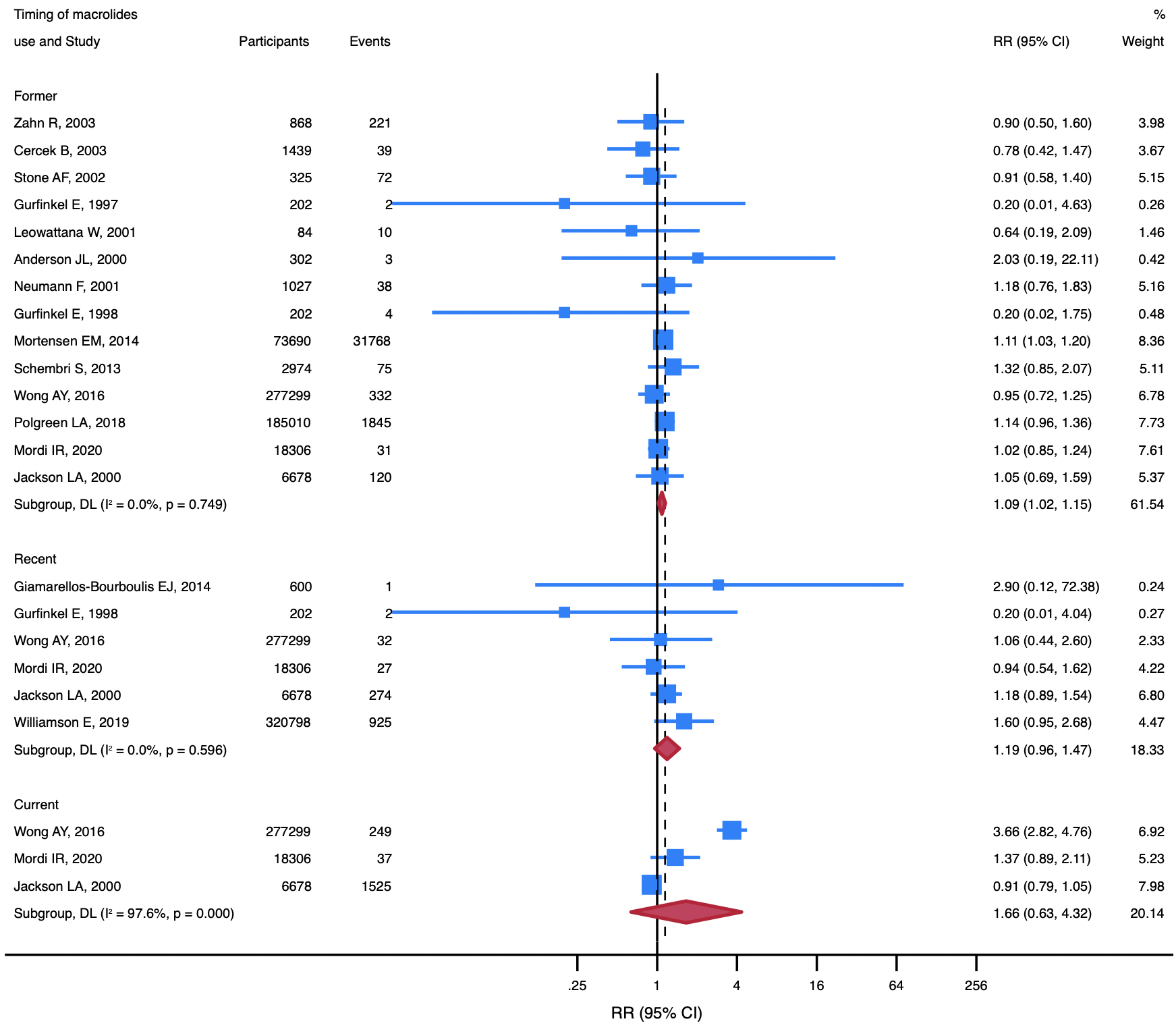


**Supplemental Figure 6**. Timing of macrolides use subgroup analysis showing RR of MI. **Squares** represent mean values, with the size of the squares indicating weight and **horizontal lines** representing 95% CIs. The **diamond** represents the pooled mean with the points of the diamond representing 95% CIs. **RR** = relative risk; **CI** = confidence interval; **MI** = myocardial infarction. **Current** referred to the patient's current use of macrolides, **recent** was defined as use of macrolides within one month, and **former** involved the use of macrolides within one year.


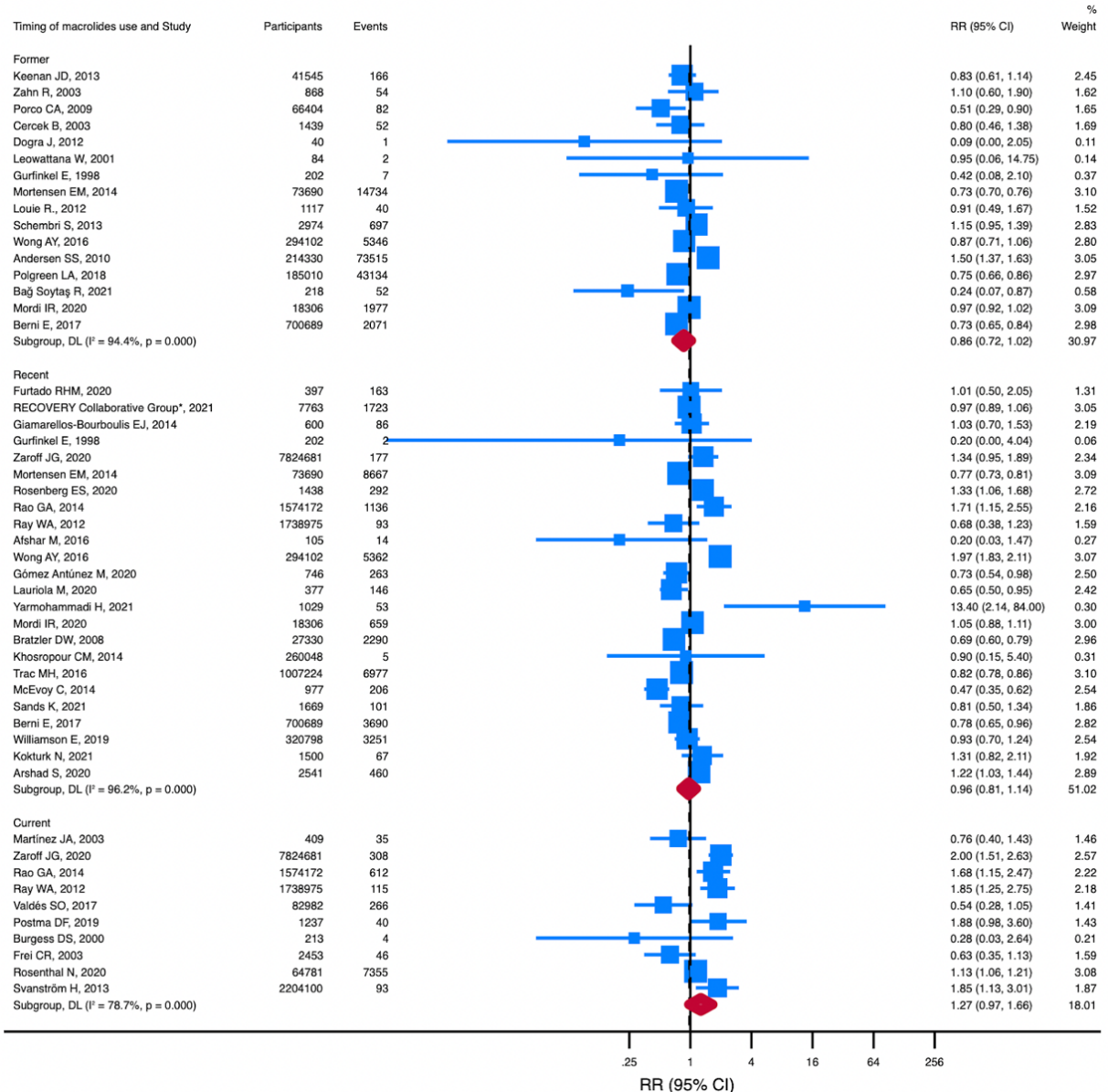


**Supplemental Figure 7**. Timing of macrolides use subgroup analysis showing RR of all-cause death. **Squares** represent mean values, with the size of the squares indicating weight and **horizontal lines** representing 95% CIs. The **diamond** represents the pooled mean with the points of the diamond representing 95% CIs. **RR** = relative risk; **CI** = confidence interval. **Current** referred to the patient's current use of macrolides, **recent** was defined as use of macrolides within one month, and **former** involved the use of macrolides within one year.


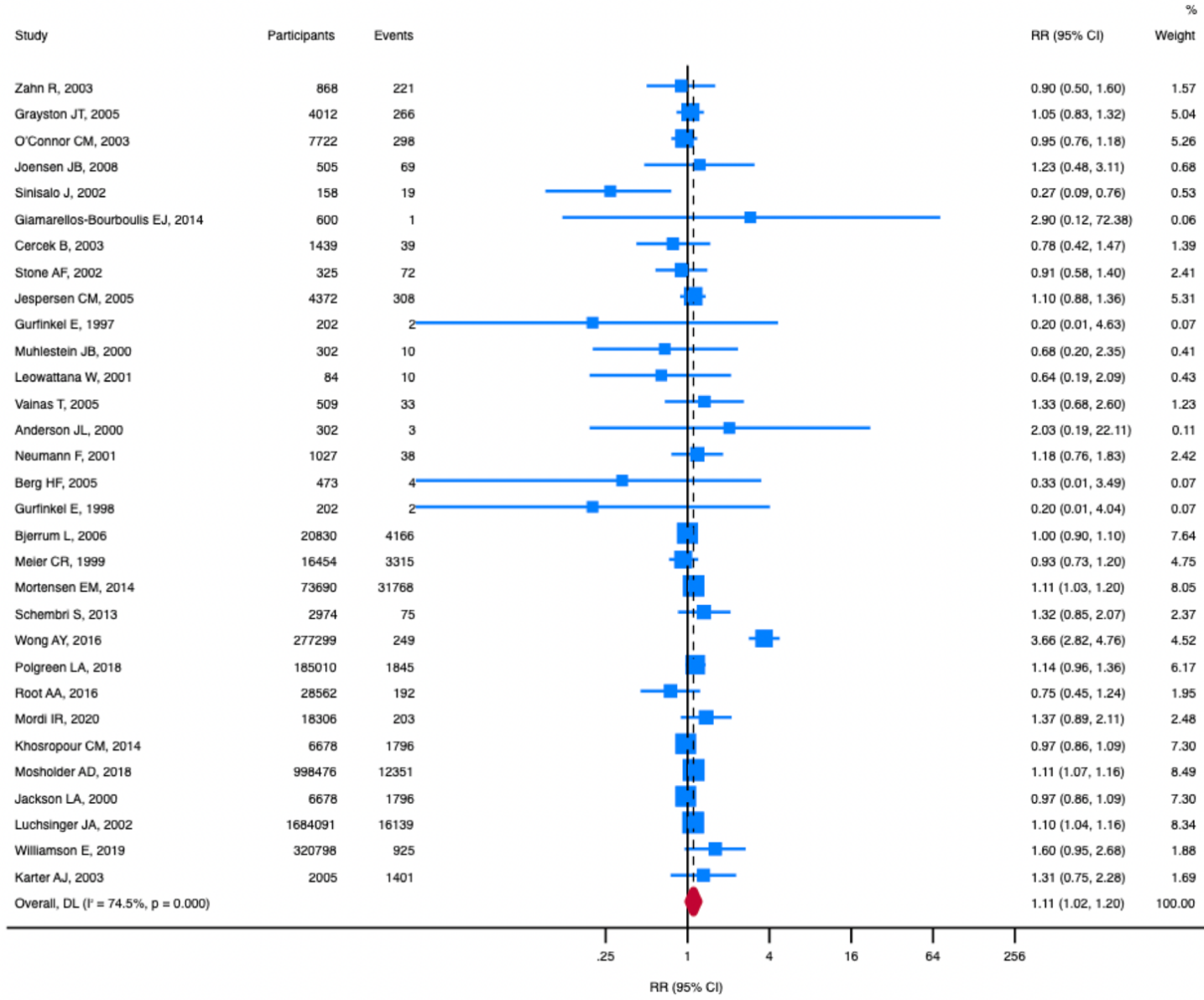


**Supplemental Figure 8**. RR of MI. **Squares** represent mean values, with the size of the squares indicating weight and **horizontal lines** representing 95% CIs. The **diamond** represents the pooled mean with the points of the diamond representing 95% CIs. **RR** = relative risk; **CI** = confidence interval; **MI** = myocardial infarction.

**
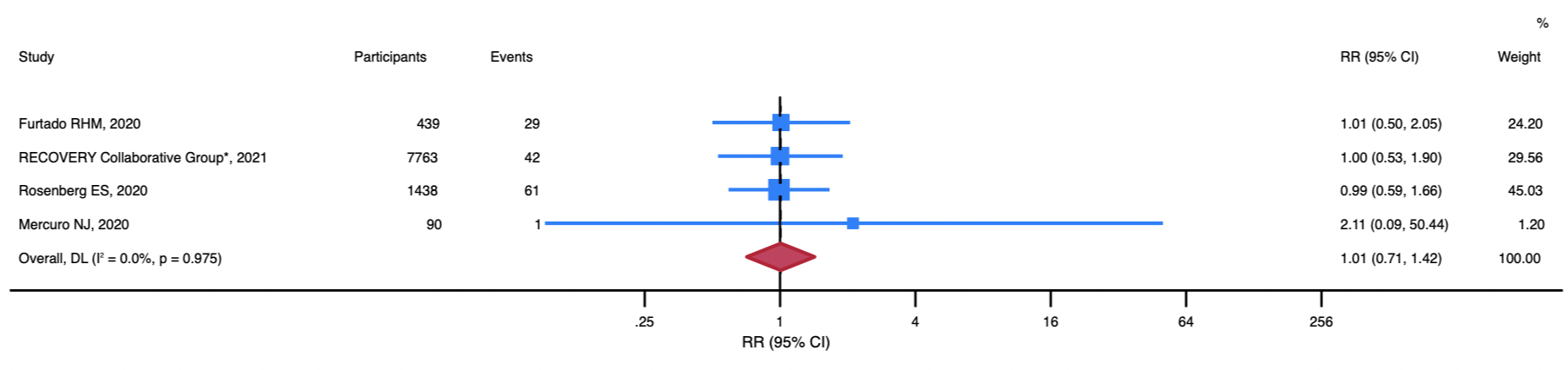
**

**Supplemental Figure 9**. Macrolides and VA or SCD in patients with COVID-19. **Squares** represent mean values, with the size of the squares indicating weight and **horizontal lines** representing 95% CIs. The **diamond** represents the pooled mean with the points of the diamond representing 95% CIs. **RR** = relative risk; **CI** = confidence interval; **VA or SCD** = ventricular arrhythmia or sudden cardiac death.


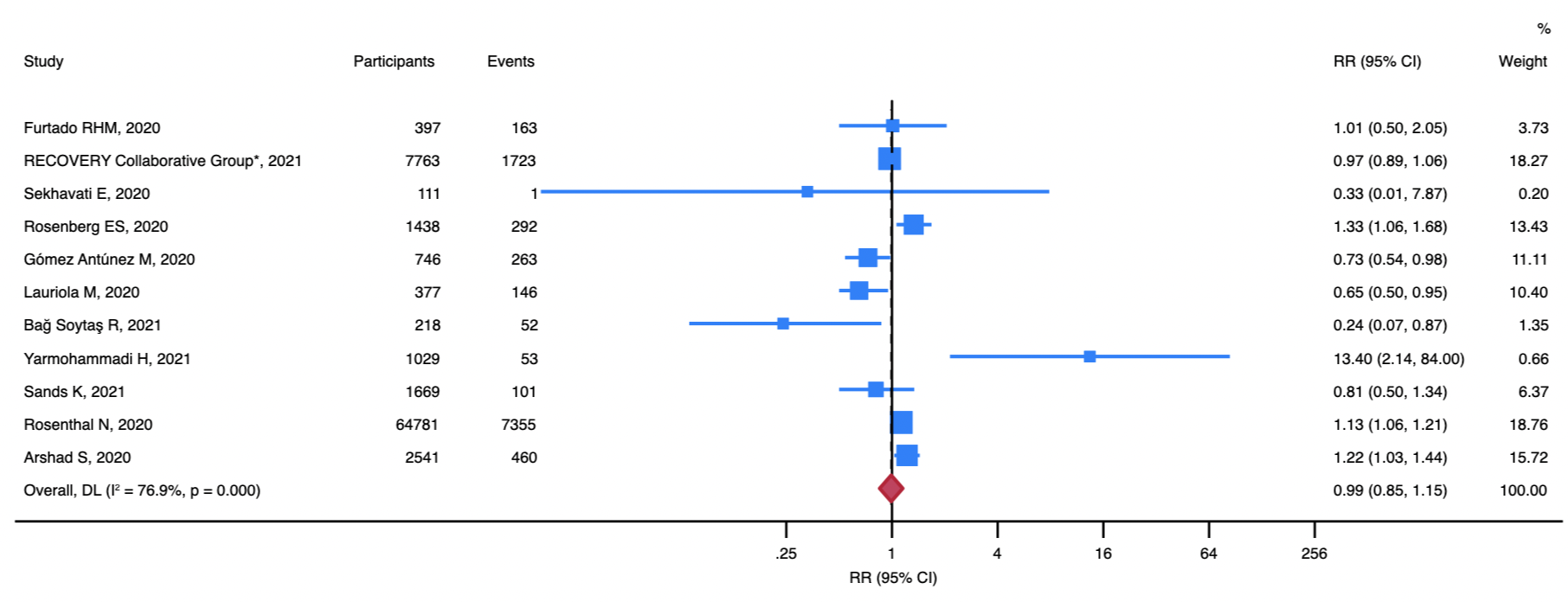


**Supplemental Figure 10**. Macrolides and all-cause death in patients with COVID-19. **Squares** represent mean values, with the size of the squares indicating weight and **horizontal lines** representing 95% CIs. The **diamond** represents the pooled mean with the points of the diamond representing 95% CIs. **RR** = relative risk; **CI** = confidence interval.
